# Biosynthesis of plant tetrahydroisoquinoline alkaloids through an imine reductase route†
†Electronic supplementary information (ESI) available. See DOI: 10.1039/c9sc03773j


**DOI:** 10.1039/c9sc03773j

**Published:** 2019-11-18

**Authors:** Lu Yang, Jinmei Zhu, Chenghai Sun, Zixin Deng, Xudong Qu

**Affiliations:** a State Key Laboratory of Microbial Metabolism , School of Life Sciences and Biotechnology , Shanghai Jiao Tong University , Shanghai 200240 , China . Email: quxd@whu.edu.cn; b Key Laboratory of Combinatorial Biosynthesis and Drug Discovery Ministry of Education , School of Pharmaceutical Sciences , Wuhan University , Wuhan 430071 , China

## Abstract

Herein, we report a biocatalytic approach to synthesize plant tetrahydroisoquinoline alkaloids (THIQAs) from dihydroisoquinoline (DHIQ) precursors using imine reductases and *N*-methyltransferase (NMT). The imine reductase IR45 was engineered to significantly expand its substrate specificity, enabling efficient and stereoselective conversion of 1-phenyl and 1-benzyl 6,7-dimethoxy-DHIQs into the corresponding (*S*)-tetrahydroisoquinolines (*S*-THIQs). Coclaurine *N*-methyltransferase (CNMT) was able to further efficiently convert these (*S*)-THIQ intermediates into (*S*)-THIQAs. By assembling IRED, CNMT, and glucose dehydrogenase (GDH) in one reaction, we effectively constituted two artificial biosynthetic pathways in *Escherichia coli* and successfully applied them to the production of five (*S*)-THIQAs. This highly efficient (100% yield from DHIQs) and easily tailorable (adding other genes) biosynthetic approach will be useful for producing a variety of plant THIQAs.

## Abstract 2

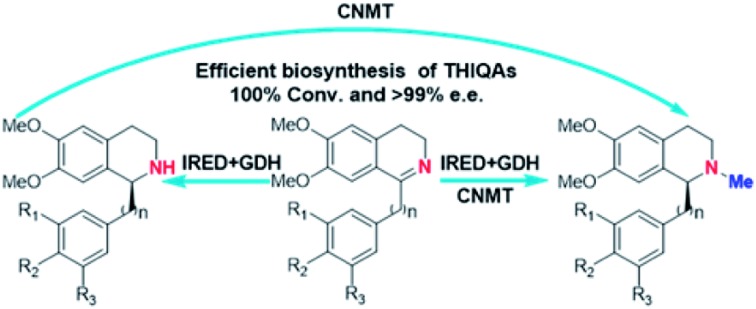
An artificial approach has been developed for efficient biosynthesis of plant tetrahydroisoquinoline alkaloids from dihydroisoquinoline precursors.

## Introduction

**Scheme 1:**
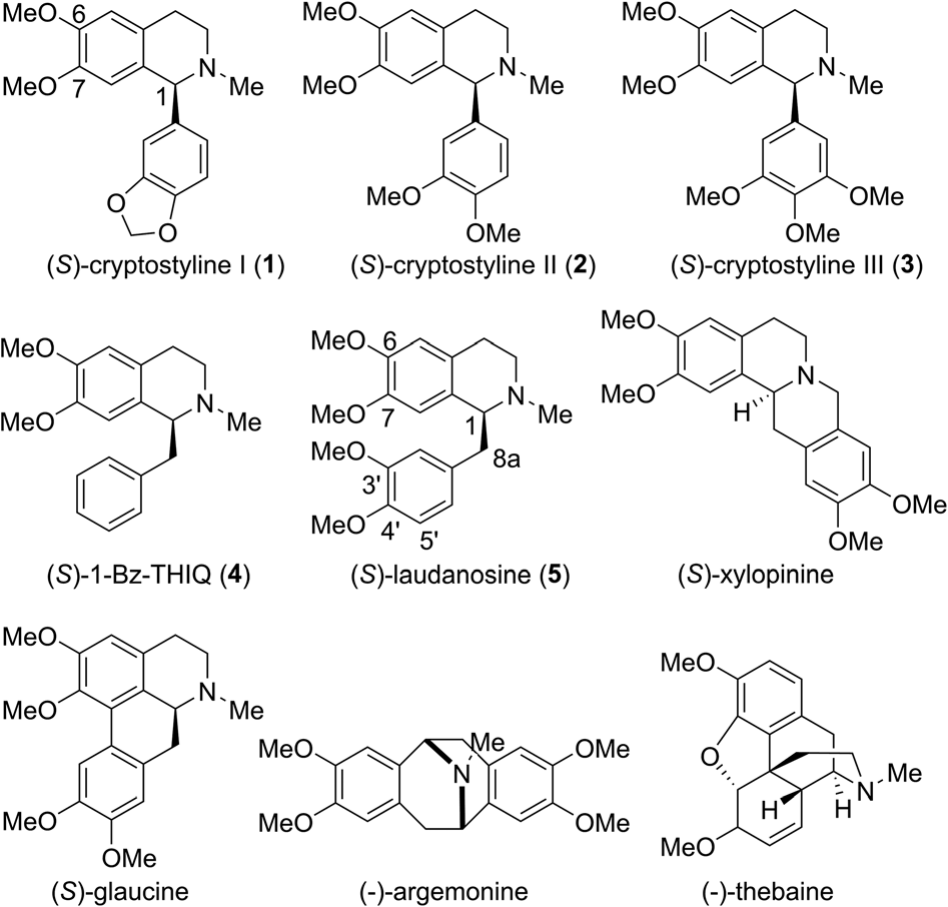
Representative THIQAs.

Tetrahydroisoquinoline alkaloids (THIQAs) are a class of important bioactive natural products.^1^ Molecules in this family typically contain benzyl (benzylisoquinoline alkaloids, BIAs) or phenyl (phenylisoquinoline alkaloids, PIAs) groups at the C-1 position and show a remarkable spectrum of biological activity (Scheme 1).^1^,^2^ Currently, more than a dozen of naturally occurring or semisynthetic THIQAs have been used in the clinic for treating a diverse range of diseases.^1^–^3^ As most THIQAs are produced by plants, significant efforts have been devoted in the past few decades to develop synthetic approaches for their preparation.^1^ However, the unsatisfactory enantioselectivity and high cost of asymmetric catalysis make chemical approaches still commercially unfeasible.^1^

**Scheme 2:**
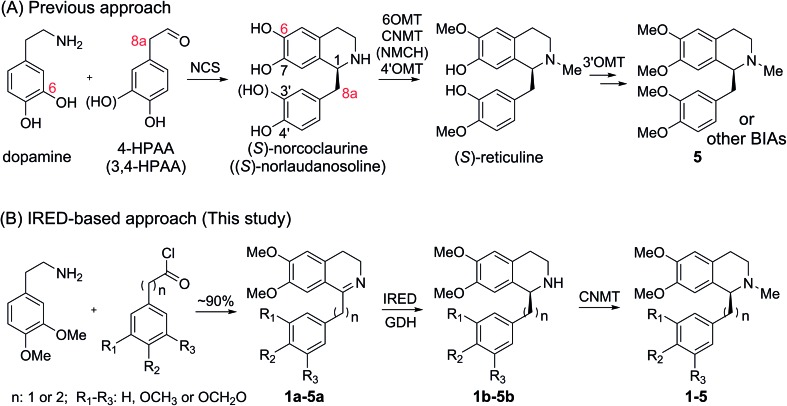
Approaches for plant THIQA biosynthesis.

In natural products, THIQ scaffolds are biosynthesized through a Pictet–Spengler reaction.^4^ The major enzymes which catalyze these reactions are norcoclaurine synthases (NCSs). NCSs condense dopamine with 4-hydroxyphenylacetaldehyde (4-HPAA) or 3,4-hydroxyphenylacetaldehyde (3,4-HPAA) to give rise to (*S*)-norcoclaurine or (*S*)-norlaudanosoline.^5^ Following decorations by methyltransferases (MTs) or P450s, 4-HPAA and 3,4-HPAA can be converted into (*S*)-reticuline (Scheme 2), a key intermediate of ∼2500 BIAs.^5^ With the growing understanding of BIA biosynthesis, reconstruction of plant biosynthetic pathways in microbial hosts has become a feasible strategy for producing BIAs. By incorporation of partial or complete biosynthetic pathways in microbial hosts, a number of BIAs (*i.e.* opiates, sanguinarine and noscapine) have been successfully synthesized from simple precursors or glucose.^6^ Such biosynthetic approaches can become more economical and easy-to-operate than chemical synthesis, making biosynthesis a highly attractive alternative.

Although great success has been achieved, there still remain many challenges for microbial production of plant THIQAs:^7^ (i) the biosynthesis of PIAs and many BIAs remain elusive, and thus, knowledge of essential genes for reconstitution of their biosynthetic pathways is lacking. (ii) Most valuable THIQAs are highly modified, *i.e.* bearing methoxy groups at the C6, C7, C3′ and C4′ positions. (iii) Reconstitution of the biosynthetic pathways in microbes requires the introduction of a large numbers of genes, which brings a heavy metabolic burden on the microbe. Moreover, many plant enzymes such as OMTs and P450s are poorly expressed or show low reactivity (NCSs) in microbial systems.6e,k,^8^ Therefore, production of plant THIQAs in microbes often results in very low yields.^3^ To avoid these challenges, appropriately substituted precursors can be fed into simplified biosynthetic pathways. However, such an approach is restricted either by the narrow substrate scope of NCSs, whose activity is strictly dependent on the C_6_-hydroxyl and C_8a_-methylene groups (Scheme 2)^9^ or the stereochemistry at C1, which is challenging to prepare *via* practical chemical synthesis.^1^

Recently, our lab and another group identified imine reductases (IREDs) that are capable of reducing sterically hindered 1-phenyl dihydroisoquinolines (DHIQs) to yield the corresponding chiral 1-phenyl-THIQs.^10^ In contrast to NCSs, IREDs have a distinct catalytic mechanism, which does not rely on the C_6_-hydroxyl or C_8a_-methylene group, and therefore can accept highly modified DHIQ precursors. Moreover, IREDs from bacterial sources can be well overexpressed and show good activity in *Escherichia coli.* Thus, by combining IREDs with other modification enzymes, it is potentially feasible to produce THIQAs through feeding DHIQ precursors. Herein, we demonstrate this concept by using IREDs, *N*-methyltransferases (NMTs), and glucose dehydrogenase (GDH) to construct two minimal biosynthetic pathways. By incorporating these artificial pathways into *E. coli*, we successfully synthesize a group of pharmaceutically valuable PIAs and BIAs. Our results demonstrate that this strategy is an efficient approach to the biosynthesis of plant THIQAs.

## Results

### Engineering the substrate specificity of IR45 for the synthesis of 1-phenyl 6,7-dimethoxyl-THIQs

Cryptostyline I, II and III (**1–3**, Scheme 1) are three PIAs isolated from the plant *Cryptostylis fulva*.^11^ They are used as pharmacological probes for understanding the pathophysiological roles of peptides in the nervous system.^12^ Unlike BIAs, the biosynthesis of PIAs is still poorly understood, and currently the biosynthetic genes of **1–3** are not known. Given their valuable pharmaceutical utility and representative roles for the PIAs, these three products were chosen as the targets for this study.

**Scheme 3:**
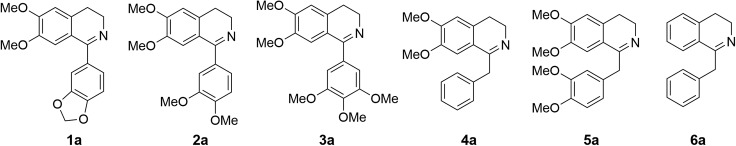
DHIQ substrates prepared and used in this study.

**Fig. 1:**
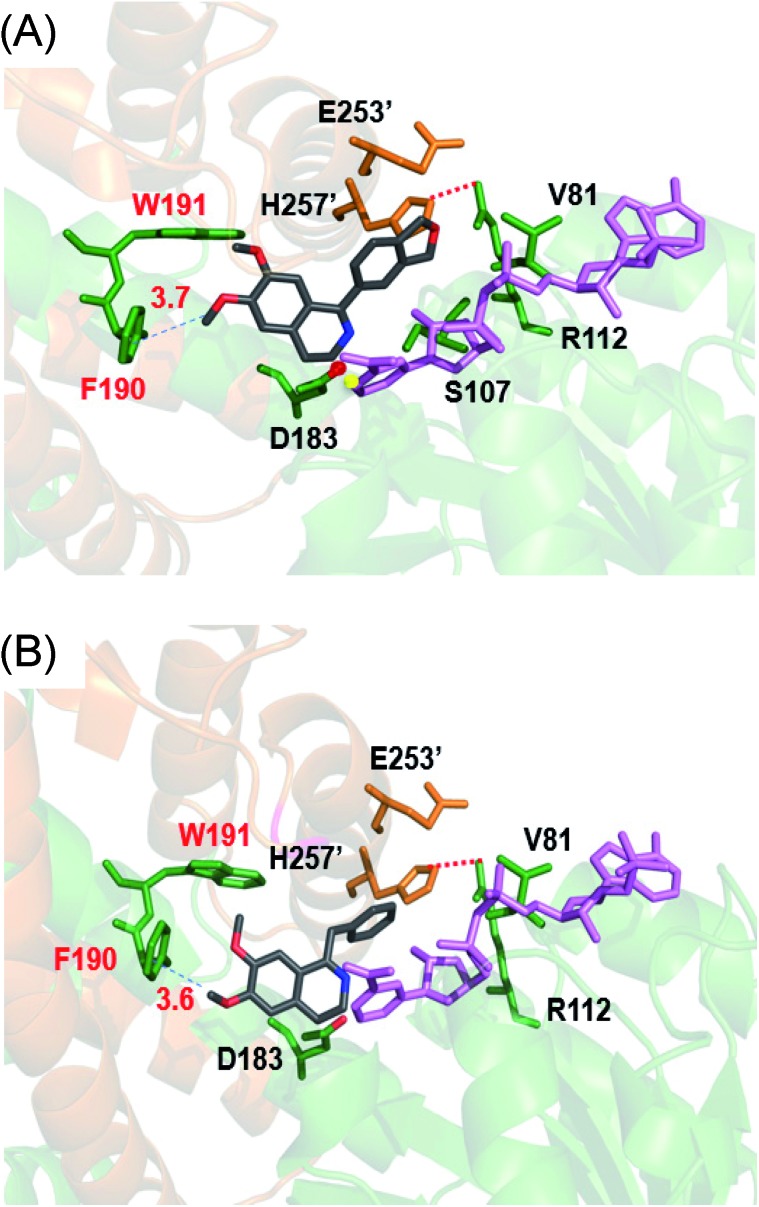
*In silico* model of IR45 with substrates **1a** and **4a**. The structure of IR45 is modelled based on Q1EQE0 (PDB: ; 3zhb) and docked with **1a** or **4a** (grey) and NADPH (pink); critical residues in the binding cavity are labelled. Protein backbones and residues from different subunits are indicated by yellowish brown and green colours. (A) IR45 with **1a**; (B) IR45 with **4a**.

Three 1-phenyl-6,7-dimethoxy-DHIQ precursors (**1a–3a**, Scheme 3) corresponding to cryptostyline I–III (**1–3**) were obtained in good yields (90–91%) by chemical synthesis (see the ESI†). In a previous study, we identified an *S*-selective IRED, IR45, which showed good tolerance toward 1-phenyl-DHIQs.10*a* Therefore, this enzyme was selected for activity assays with the new substrates. Encouragingly, IR45 effectively reduced **1a** to the (*S*)-1-methylenedioxyphenyl-6,7-dimethoxy-THIQ (**1b**, Scheme 2) with 100% conversion and >99% ee (Table 1), although it showed no activity toward the two bulkier substrates (**2a** and **3a**). To gain a more detailed understanding of its substrate specificity, the structure of IR45 was modelled based on the IRED Q1EQE0 (64% identity, PDB: ; 3ZHB).^13^ Docking of **1a** into the binding pocket of IR45 indicated that the substrate is closely surrounded by the conserved aspartic acid (D183) and NADPH (Fig. 1). The imine bond is positioned close to the *si*-face of NADPH, consistent with the observed *S*-selectivity in the product. The methylenedioxyphenyl group points to the right cleft, which consists of residues from two subunits of the enzyme (V81, S107, E253′ and H257′). On the other side of the active site, the 6,7-dimethoxy aryl group is surrounded by two residues, W191 and F190. These two bulky residues form the bottom of the binding pocket, with F190 being the major contributor to the pocket's overall shape.

**Table 1:** Conversion and enantioselectivity of IR45 and its mutants toward **1a–5a**

Subs.	Conversion (%) and enantiomeric excess (ee)^*a*^ (%)			
	IR45	W191F	F190L–W191F	F190M–W191F
**1a**	100; >99^*S*^	—	100; >99^*S*^	100; >99^*S*^
**2a**	0	3; —	75; >99^*S*^	0
**3a**	0	2; —	100; >99^*S*^	0
**4a**	100; >99^*S*^	—	100; >99^*S*^	100; >99^*S*^
**5a**	100; >99^*S*^	—	100; >99^*S*^	100; >99^*S*^

^*a*^First value shown represents conversion and the second value represents the ee value. Stereospecificity is indicated by the superscripts. Dash indicates data not tested. All reactions were performed at 30 °C for 24 h.

From the model, it can be seen that the right cleft forms the major constraint for the accommodation of the methylenedioxyphenyl group (Fig. 1). Engineering this area, however, may significantly impair the enzyme's activity and stability, as residues in this region are crucial for inter-subunit interactions, *i.e.* H257′ forms a stabilizing salt bridge with R112. Thus, we envisioned enlarging the binding pocket by engineering the side of the pocket comprising F190 and W191. By mutating these sterically demanding residues, we thought that the substrate could be moved slightly deeper into the left cavity, thus potentially relieving the steric hindrance between the right cleft and substrates **2a** and **3a**.

In our previous studies, we found that W191 plays important roles in substrate binding and maintaining the enzyme's catalytically active conformation. Mutation of W191 to alanine or leucine reduces the *k*_cat_ and *K*_m_ for the substrate 1-phenyl-DHIQ (**6a**, Scheme 3).10*a* To identify optimal mutations, W191 was further mutated to the remaining 17 amino acids. These variants together with the W191A and W191L were assayed for the reduction of **2a** and **3a**. Although most mutants were inactive, one mutant in particular, W191F, showed marginal but clear activity toward these substrates (3% and 2% for **2a** and **3a** respectively, Table 1 and Fig. S1A†). To unveil the detailed effects of these mutations on the IRED's activity, we used the more active substrate **6a** for activity analysis. Similarly, W191F shows a clear improvement in the conversion of 1-phenyl-DHIQ (1.5-fold), while the others all drastically reduce or even abolish the catalytic activity (Fig. S2†).

Kinetic analysis of the most active mutants, including W191F, W191C, W191M, W191H, W191N, and W191S as well as previously obtained W191A and W191L revealed that their *k*_cat_ and *K*_m_ values both have been reduced (Table S1 and Fig. S3A†). However, unlike the other enzymes whose *k*_cat_ is significantly reduced by one to two orders of magnitude, the *k*_cat_ value of W191F is decreased by only 1.8-fold (Table S1†), suggesting that W191F has the least negative impact on the enzyme's catalytic conformation. As W191 is highly conserved, we assume that this mutation should be beneficial for many other IREDs.

The encouraging results with W191F inspired us to further engineer the enzyme by targeting the adjacent residue F190. We mutated all 19 other residues on top of W191F and then assayed these double mutants for activity on the bulkier DHIQ substrates. To our gratification, one mutant F190L–W191F shows drastically improved activity toward both **2a** and **3a** (75% and 100% conversion within 24 hours respectively) with very high stereoselectivity for the *S*-products **2b** and **3b** (Scheme 2 and Table 1). Kinetic analysis confirmed that both **2a** and **3a** are good substrates for F190L–W191F (*k*_cat_/*K*_m_ values are 0.011 and 0.087 s^–1^ mM^–1^, respectively, Table 2). The identical *K*_m_ values (0.307 mM for **2a** and 0.372 mM for **3a**) yet different *k*_cat_ values (0.003 s^–1^ for **2a** and 0.032 s^–1^ for **3a**) suggest that binding conformation accounts for the difference in their activity. This indicates that other residues influencing the binding conformation in the cavity could be engineered to further improve the enzyme's catalytic activity.

**Table 2:** Kinetics parameters of IR45 and its mutants for the conversion of **1a–5a**^*a*^

Subs.	IR45			F190L–W191F			F190M–W191F		
	*K*_m_ [mM]	*k*_cat_ [s^–1^]	*k*_cat_/*K*_m_ [s^–1^ mM^–1^]	*K*_m_ [mM]	*k*_cat_ [s^–1^]	*k*_cat_/*K*_m_ [s^–1^ mM^–1^]	*K*_m_ [mM]	*k*_cat_ [s^–1^]	*k*_cat_/*K*_m_ [s^–1^ mM^–1^]
**1a**	0.154	0.020	0.129	0.123	0.009	0.076	0.108	0.031	0.288
**2a**	NA	NA	NA	0.307	0.003	0.011	NA	NA	NA
**3a**	NA	NA	NA	0.372	0.032	0.087	NA	NA	NA
**4a**	0.148	0.021	0.146	0.252	0.049	0.196	0.251	0.248	0.989
**5a**	0.518	0.090	0.174	0.353	0.041	0.117	0.178	0.054	0.306

^*a*^NA indicates no activity.

Through protein engineering, we have successfully broadened the substrate specificity and enabled IR45 to effectively convert very sterically hindered DHIQ precursors into their corresponding THIQs. In addition, the successful expansion of the substrate specificity of IR45 demonstrates that alteration of the sites that do not directly repel the substrate is indeed an effective way to optimize an enzyme's substrate specificity. This strategy will be useful for engineering other proteins, in which certain substrate–protein interactions are critical for maintaining catalytic activity or active conformation.

### Employing IR45 mutants to synthesize 1-benzyl 6,7-dimethoxyl-THIQs

Because BIAs make up the remainder of the THIQA family, we were interested in applying IR45 to their synthesis as well. (*S*)-Laudanosine (**5**, Scheme 1) is a pharmaceutically important BIA, naturally found in opium in minute amounts (0.1%).^14^ Its semisynthetic derivative atracurium is medically used for skeletal muscle relaxation during surgery and mechanical ventilation.^3^ By mild chemical oxidation, (*S*)-laudanosine can be readily converted into the antitussive drug (*S*)-glaucine.^15^ Production of racemic (*R*,*S*)-laudanosine in *E. coli* has been previously achieved through feeding (*R*,*S*)-norlaudanosoline to a strain expressing O-MTs and NMTs.^16^ However, the poor activities of O-MTs result in low yields of (*R*,*S*)-laudanosine, and the accumulation of many methylated intermediates (*e.g.* (*R*,*S*)-reticuline, (*R*,*S*)-codamine and (*R*,*S*)-laudanine) complicates its isolation (Fig. S4†). We chose to test our IRED approach in the synthesis of **5** given its pharmaceutical value and (*S*)-1-benzyl-6,7-dimethoxyl-*N*-methyl-THIQ (BzTHIQ, **4** in Scheme 1) given its prototypical structure.

Two corresponding DHIQ substrates **4a** and **5a** were chemically synthesized (yields 95 and 93%) following a procedure similar to that used in the synthesis of **1a–3a**. As W191 and F190 are critical for substrate binding, wild-type IR45 along with the library of F190X–W191F (X denotes other 19 amino acids) variants was tested for activity. To our gratification, wild-type IR45 effectively converted **4a** and **5a** into the corresponding (*S*)-THIQ products (100% conversion and >99% ee). The best mutant F190M–W191F showed a further 6.8- and 1.8-fold improvement in catalytic activity for **4a** and **5a**, respectively (*k*_cat_/*K*_m_ values for **4a** and **5a** are 0.989 and 0.306 s^–1^ mM^–1^, Table 2).

It is intriguing that the wild-type IR45 can accept **4a** and **5a** whose planar frameworks are larger than those of the inactive substrates **2a** and **3a**. To explore how differences in binding in the enzyme cavity may account for this disparity in activity, we docked **4a** into IR45 (Fig. 1B). *In silico* analysis revealed that the DHIQ plane of **4a** takes an identical conformation to that of **1a**. On the other hand, the benzyl group of **4a** is bent toward the large cavity underneath the right cleft unlike the 1-phenyl group of **1a** which points directly towards the right cleft. This difference in positioning may result in less steric hindrance for the otherwise ‘larger substrate’ **4a**. This indicates that the rigid frameworks of 1-phenyl DHIQs are indeed the most challenging substrates for IREDs. Fortunately, our engineered mutant F190L–W191F is able to effectively convert the very sterically hindered substrates **2a** and **3a**. In addition to the elevated activity on **4a** and **5a**, F190M–W191F also shows a 2.23- and 3.79-fold improvement (*k*_cat_/*K*_m_) on **1a** compared to wild-type IR45 and F190L–W191F, respectively (Table 2). This outstanding activity makes the imine reduction step highly efficient, ensuring the success of this strategy.

### Conversion of THIQs into THIQAs by CNMT

After overcoming the bottlenecks associated with imine reduction, we turned our attention to the *N*-methylation step. Coclaurine *N*-methyltransferase (CNMT) is a key enzyme in the pathway of (*S*)-reticulene, introducing the *N*-methyl substituent into coclaurine.^17^ CNMT from *Coptis japonica* is known to have a relaxed substrate specificity and is able to methylate THIQ products.^17^,^18^ Therefore, it was assayed for *N*-methylation activity on THIQ substrates **1b–5b**. To improve expression, the CNMT gene was codon optimized for *E. coli*. As expected, following optimization, CNMT was robustly expressed in *E. coli*.

To our gratification, CNMT efficiently converted all five of the *S*-THIQ substrates into their corresponding *N*-methylated products (100% conversion, Fig. S5A†). Kinetic analysis revealed that CNMT showed similar activity to all 1-phenyl and 1-benzyl substrates except for the most active substrate **4b** (Table S2†). As these substrates are much bulkier than previously known substrates of CNMT, we were interested in understanding how they bind to the active site. Two representative substrates **1b** and **5b** were individually docked into the crystal structure of CNMT (Fig. S6†).^18^ Similar to the conformation of **4a** in IR45, **5b** is bent, allowing it to be well accommodated in the binding cavity. The substrates are bound in an identical orientation with their methylenedioxy and methoxy groups in close proximity to residues F322 and W329.^18^ Previous work showed that two mutants, F322A and W329A, had significantly reduced catalytic activity, with W329A having the most negative impact.^18^ In order to maintain these hydrophobic interactions while enlarging the binding cavity, we mutated W329 into phenylalanine. However, like W329A, W329F showed drastically reduced methylation activity toward **1–5** (Fig. S7A†), confirming that W329 is indeed critical for maintaining the enzyme's activity.

### Production of THIQAs from DHIQ precursors through the biosynthetic pathway of IRED–NMT–GDH

Armed with efficient imine reduction and *N*-methylation enzymes, we proceeded to combine these two steps into one biocatalytic reaction. The purified F190L–W191F and F190M–W191F were coupled with CNMT and glucose dehydrogenase (GDH, for recycling NADPH from glucose) for the synthesis of **2–3** and **1**, **4–5** respectively. As expected, these enzyme cascades can efficiently convert **1a–5a** into THIQAs with 100% conversion (within 24 h, Fig. S7B†). Moreover, we observed that *N*-methylation improved the conversion of substrates **2a** and **3a** into their corresponding THIQAs. We hypothesize that the continuous removal of THIQ intermediates by their irreversible methylation drives the equilibrium of imine reduction from DHIQs to THIQs.

With the encouraging results of *N*-methylation, we turned to constitution of the artificial biosynthetic pathway in *E. coli*. To achieve the highest transformation efficiency, each step was evaluated individually in the *E. coli* system. We first evaluated the effect of temperature and IPTG concentration on the IRED reaction. *E. coli* stains containing F190M–W191F and GDH (cloned into pET28a and pACYCDuet, respectively) were incubated at 25, 30 or 37 °C and supplemented with **1a** and different concentrations of IPTG when their OD_600_ reached 0.7. The biotransformation results show that lower IPTG dosage (20 μM) is better than a high IPTG dosage (Fig. S8A†) with conversion reaching only 70% and 40% with 50 μM and 100 μM IPTG, respectively, after 3 days. We found that temperature also had a significant impact on the conversion. The biotransformation efficiency at 30 °C is 5 and 10 times higher than that at 25 °C and 37 °C, respectively (after 3 days) (Fig. S8B†). The optimal conditions for the IRED–GDH combination appear to be 20 μM IPTG and 30 °C. Next the conversion of the other four substrates was evaluated under these optimized conditions (F190M–W191F was replaced by F190L–W191F for **2a** and **3a**). Although **4a** and **5a** can be efficiently converted into their reduced products (100%), **1a–3a** exhibit poor-to-medium conversion (6.5% to 50%) (Fig. S1C†). These ratios are much lower than the conversion ratios of the *in vitro* enzymatic transformation, suggesting that the 1-phenyl substrates **1a–3a** face difficulty in permeating the cell membrane.

**Fig. 2:**
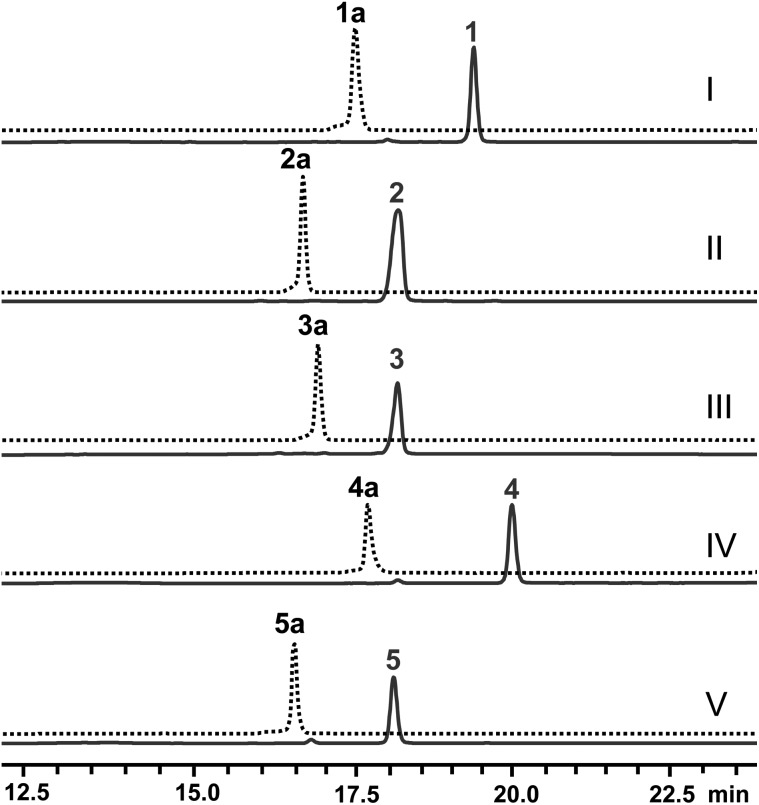
Production of THIQAs **1–5** in the IRED-based biosynthetic systems. The retention time of the precursors **1a–5a** is indicated by the dashed lines. Samples from the biosynthetic systems are indicated by the solid lines in dark blue. (I) Biosynthesis of **1** from **1a** by *E. coli* with F190M–W191F + GDH + CNMT; (II) biosynthesis of **2** from **2a** by the crude enzymes F190L–W191F + GDH and *E. coli* with CNMT; (III) biosynthesis of **3** from **3a** by the crude enzymes F190L–W191F + GDH and *E. coli* with CNMT; (IV) biosynthesis of **4** from **4a** by *E. coli* with F190M–W191F + GDH + CNMT; (V) biosynthesis of **5** from **5a** by *E. coli* with F190M–W191F + GDH + CNMT.

Next, the *N*-methyl transfer step was evaluated. Gratifyingly, all the amine substrates, **1b–5b**, are completely converted into their corresponding final products under the same optimal conditions as for IRED–GDH by *E. coli* containing CNMT (Fig. S5B†). As methylation by CNMT appeared to improve the activity of imine reduction, we co-expressed all three enzymes in a single cell (NMT and GDH were cloned in pACYCDuet and IR45 in pET28a), forming a minimal biosynthetic pathway for the synthesis of THIQAs. As expected **1a**, **4a** and **5a** (50 mg L^–1^) can be completely converted into their corresponding alkaloids within one to three days (Table 3 and Fig. 2). By extraction with ethyl acetate, pure **1**, **4** and **5** can be readily obtained in yields of 95%, 98% and 95%, respectively. Conversion of **2a** and **3a** also significantly improved (89% and 51% respectively, Fig. S7C†), compared to the conversion by IRED–GDH (6.5% and 9.3%, Fig. S1C†).

**Table 3:** Biosynthesis of **1–5** from precursors **1a–5a** through the IRED-based pathways

Subs.	Enzymes	Time (days)	Conv. (%)
**1a**	F190M–W191F + GDH + CNMT	3	100^*a*^
**2a**	F190L–W191F + GDH + CNMT	3	100^*b*^
**3a**	F190L–W191F + GDH + CNMT	3	100^*b*^
**4a**	F190M–W191F + GDH + CNMT	1	100^*a*^
**5a**	F190M–W191F + GDH + CNMT	3	100^*a*^

^*a*^Whole cell biosynthesis.

^*b*^Enzyme-cell biosynthesis.

In order to overcome the barrier of membrane permeabilization and further improve the conversion of **2a** and **3a**, we combined enzymatic and whole-cell biotransformation into a single pot reaction. F190L–W191F and GDH were first overexpressed in a single *E. coli* strain and then lysed by sonication. The crude enzymes were directly added to the *E. coli*–CNMT culture when the OD_600_ reached 0.7. At this time, IPTG, DHIQ substrates and NADP (1.1‰ w/v) were added simultaneously to initiate the CNMT expression and cascade biotransformation. To our delight, both **2a** and **3a** (50 mg L^–1^) were completely converted into their corresponding THIQAs (Table 3 and Fig. 2). The isolated yields of **2** and **3** are 93% and 96%, respectively. Therefore, by systematically improving enzyme activity and optimizing biocatalytic conditions, we successfully implemented the IRED-based route to biosynthesize two types of plant THIQAs.

## Discussion

Lack of knowledge of key biosynthetic genes and poor expression/low reactivity of plant biosynthetic enzymes make reconstitution of THIQA biosynthetic pathways in microbes very challenging. Currently, the biosynthetic enzymes, including the key Pictet–Spengler synthase, that assemble PIAs remain unknown. As such, reconstitution of PIA biosynthesis in microbes is yet to be realized. Although the biosynthesis of many BIAs is well understood and elegant examples of their production in microbes have been reported, the poor performance of NCS O-MT and P450 enzymes results in overall low yields.^3^ Efforts to bypass such bottleneck enzymes by employing modified precursors are also limited by the narrow substrate scope of NCSs.^9^ IREDs, owing to their distinct catalytic mechanism, are not restricted by the same limitations as NCSs. Therefore, bottleneck enzymes such as O-MTs and P450 hydroxylases can be omitted and replaced by readily available synthetic DHIQ precursors. In conjunction with NMT and/or additional other modification enzymes, this IRED-based approach has been shown to be very powerful in the biosynthesis of plant THIQAs.

The major obstacle for application of this approach is the generally poor tolerance of IREDs to bulky substituents at C1 of DHIQs. IR45 is a steric-hindrance tolerating IRED identified in our previous study.10*a* Due to their more rigid conformations, 1-phenyl 6,7-dimethoxyl-DHIQ substrates are more sterically hindered than the 1-benzyl 6,7-dimethoxyl-DHIQs, and wild-type IR45 showed no activity on the most sterically hindered substrates **2a** and **3a**. To address this issue, we engineered IR45 to significantly expand its substrate scope. Instead of engineering the site of direct steric repulsion, we chose to alter the other side of the binding pocket. This strategy was very effective for IR45 and may prove to be useful for engineering other enzymes where certain protein interactions nearby the substrate are critical for maintaining catalytic activity or conformation.

The resultant mutant W191F–F190 efficiently and stereo-selectively (ee > 99%) converted the very sterically hindered substrates (**2a** and **3a**) and the other three substrates (**1a**, **4a** and **5a**) into THIQs. The other mutant F190M–W191F showed significantly improved activity toward **1a**, **4a** and **5a**. As these substrates are more sterically hindered than most other natural PIAs and BIAs,^1^,^2^ these IR45 mutants are potentially useful for the synthesis of many other THIQAs. Practical application of these enzymes can be backed up by the recently designed strategy for scaling up IRED transformation.^19^ In addition to the IRED, we also found that the CNMT has a sufficiently relaxed substrate specificity to accept very bulky THIQ substrates. Therefore, the broad substrate specificity of both the IR45 mutants and CNMT has provided a solid basis for the application of this IRED-based biosynthetic strategy.

Although most natural THIQAs contain *S*-configuration at the C1 position, it is still interesting to generate *R*-type THIQAs using this strategy. However, our attempt to produce *R*-**1b–5b** using the most broad-selective R-type imine reductase IR2 (ref. 10*a*) was not successful. Like IR45, IR2 shows *S*-specificity to convert **1a**, **4a** and **5a** into the corresponding *S*-THIQs (**2a** and **3a** are inactive, data not shown). The reverse stereo-specificity might be due to the occurrence of the 6,7- and 3′,4′-substituent groups in the THIQ scaffold. Currently, protein engineering of IR2 is in progress to reverse its stereo-specificity.

Finally, to accommodate the different physical properties of PIAs and BIAs, we developed two types of biocatalytic approaches for their synthesis. BIAs can be biosynthesized (100% yield) from their DHIQ precursors using the recombinant *E. coli* strain expressing the artificial biosynthetic pathway. Due to the poor membrane-permeability of their DHIQ precursors, PIAs **2** and **3** were not produced by a traditional whole-cell system in sufficiently high yields. To overcome this challenge and avoid using the expensive *S*-adenosylmethionine (SAM) cofactor in the *N*-methylation step, we conceived a novel biocatalytic approach combining the enzymatic transformation of IRED–GDH and the whole-cell transformation of CNMT in one pot. This chimeric system is as efficient as the cell-based system (produces PIAs in 100% yield) and will be useful for biosynthesis of other products with similar problems.

## Conclusions

We have developed an IRED-based strategy to successfully biosynthesize five pharmaceutically valuable PIAs and BIAs. Notably, the three PIAs' natural biosynthetic pathways have not been elucidated. Through protein engineering, we significantly expanded the substrate specificity of the IRED IR45. The two resultant mutants F190L–W191F and F190M–W191F can efficiently convert bulky 1-aryl-6,7-dimethoxy-DHIQ substrates into (*S*)-THIQs. These two IREDs, highly tolerant of steric hindrance, will also be useful for the synthesis of many natural and synthetic THIQs. The *N*-methylation enzyme (CNMT) was also able to convert highly sterically hindered substrates. We also developed an efficient and cost-effective enzyme/whole-cell chimeric biosynthetic system to overcome the barrier for biotransformation of membrane-impermeable substrates. Our work demonstrates that this IRED-based biosynthetic approach is efficient (100% yield) and flexible for the production of plant THIQAs. By the addition of other modification enzymes, *e.g.* P450 enzymes, this minimal biosynthetic pathway can be further extended to the biosynthesis of other complex THIQAs.

## Conflicts of interest

The authors declare no competing financial interest.

## Supplementary Material

Supplementary information
