# Supplementary material for: Biosynthesis of plant tetrahydroisoquinoline alkaloids through an imine reductase route
Source: Chem Sci. 2019 Nov 18;11(2):364–71. doi: 10.1039/c9sc03773j (PMC7067268; doi:10.1039/c9sc03773j)
Supplement: Supplementary file 1 [file SC-011-C9SC03773J-s001.pdf]

Electronic Supplementary Material (ESI) for Chemical Science.
This journal is © The Royal Society of Chemistry 2019

Supporting Information

Biosynthesis of Plant Tetrahydroisoquinoline Alkaloids through an Imine Reductase Route

Lu Yang,[ab] Jinmei Zhu,[b] Chenghai Sun,[b] Zixin Deng,[b] and Xudong Qu*[ab]

[a]State Key Laboratory of Microbial Metabolism and School of Life Sciences and Biotechnology, Shanghai Jiao Tong University, Shanghai 200240, China.

[b]Key Laboratory of Combinatorial Biosynthesis and Drug Discovery Ministry of Education, School of Pharmaceutical Sciences, Wuhan University, Wuhan 430071, China.

Email: quxd@whu.edu.cn

Table of Contents

1. Supplementary Materials and Methods
1.1. General Materials and Methods.
1.2. Expression and Purification of Proteins.
1.3. Synthesis of Substrates.
1.4. In Vitro Biochemical Assay.
1.5. Determination of Kinetic Constants.
1.6. HPLC Analysis.
1.7. Biotransformation with Recombinant *E. coli* Strains.
2. Supplementary Tables
Table S1. Kinetic Parameters of IR45 and Its Mutants on the Conversion of **6a**.
Table S2. Kinetic Parameters of CNMT on the Conversion of **1b**-**5b**.
Table S3. Bacterial Strains and Plasmids.
Table S4. Primers Used in the Study.
Table S5. HPLC Conditions Used for Analysis of the Products.
3. Supplementary Figures
Fig. S1. HPLC analysis of imines reduction.
Fig. S2. Enzyme activity of IR45 and its mutants on the conversion of **6a** .
Fig. S3. Kinetic analysis of the IRED and CNMT reactions.
Fig. S4. Reported approach for microbial production of racemic (*R*, *S*)-laudanosine from (*R*, *S*)-norlaudanosoline.
Fig. S5. HPLC analysis of the N-methylation reactions.
Fig. S6. *Insilico* models of CNMT with the substrate **1** and **5**.
Fig. S7. HPLC analysis of the imine reduction and N-methylation reactions.
Fig. S8. Effects on the in vivo imine reduction by different IPTG concentration and temperature.
Fig. S9. 12% SDS-PAGE analysis of proteins.
Fig. S10. Chiral HPLC analysis of the enantiomeric purity of the imine products **1b**-**5b**.
Fig. S11. NMR spectrum of synthetic compounds.
4. Supplementary References

1.Supplementary Materials and Methods

1.1General Materials and Methods

Bacteria strains and plasmids are listed in Table S3. *Escherichia coli* was cultivated and manipulated according to the standard methods.[1] All chemical reactions were run under an atmosphere of argon. All other chemicals and reagents were purchased from commercial suppliers (TCI, Aldrich, Acros, Alfa and J&K etc.). Flash chromatography was performed with silica gel (200-300 mesh) from Qingdao Haiyang Chemicals. Analytical thin layer chromatography (TLC) was carried out using commercial silica-gel plates, spots were detected with UV light. Regular enzymatic conversions were analyzed in quadruplicates by a Shimazu LC-20A HPLC system with the column Diamonsil C18, 4.6 × 250 mm, 5 μm. For determining the enantiomeric excess (e.e.) of products, Chiral HPLC columns were used on an Agilent 1260 Infinity Series HPLC System. Optical rotations were measured on a polarimeter at 589 nm (sodium lamp). Proton nuclear magnetic resonance (^{1}H NMR) spectra were recorded using a Bruker AVANCE 400 (400 MHz). DNA isolation and manipulation in *E. coli* were followed standard methods. Primer synthesis and DNA sequencing were performed at Genewiz Biotech Co., Ltd. (China). Restriction enzymes and DNA polymerases (Taq and PrimeSTAR) were purchased from Takara Biotechnology Co., Ltd. (China). Protein modeling and alignment were performed by using Discovery Studio 4.0 (BIOVIA).

1.2 Expression and Purification of Proteins

Cloning and site-directed mutagenesis. The *Coptis japonica* N-methyltransferase (CNMT) gene was codon-optimized based on *E. coli* bias and synthesized by Genewiz Biotech Co. Ltd (sequence see below). It was further cloned into the *Nde*I and *Xho*I sites of the pET28a to yield the plasmid pWHU2491. Mutations in the W191 of IR45 were introduced by PCR using pWHUIR45[2] as the template and primers listed in Table S4. Mutations in the F190 of W191F were introduced by Rolling circle PCR amplification using pWHUIR45-W191F as the template. After PCR amplification 0.5 μL *Dpn*I enzyme was added to the products, and digestion was carried out at 37 °C for 2 hrs before transformation into *E. coli* DH5α. After verification by DNA sequencing,

plasmids containing mutations in W191 or F190 were further transformed into *E. coli* BL21 (DE_3) for overexpression of N-terminal 6×His-tagged fusion proteins. DNA sequence of CNMT (codon-optimized, underlined are *Nde*I and *Xho*I sites):

CATATGCAGACGAAAAAGGCGGCGATCGTTGAGCTGCTGAAGCAGCTGG
AACTGGGTCTGGTGCCGTATGACGACATCAAGCAGCTGATTCGCCGTGAA
CTGGCCCGTCGTCTGCAGTGGGGCTATAAACCGACCTACGAGGAGCAGAT
CGCGGAGATTCAGAATCTGACGCACAGTCTGCGTCAGATGAAAATCGCCA
CCGAGGTTGAGACGCTGGACAGTCAGCTCTACGAAATTCCGATTGAATTT
CTCAAGATTATGAACGGCAGCAATCTGAAGGGCAGCTGCTGTTACTTCAAG
GAAGACAGCACGACGCTGGATGAGGCCGAAATCGCCATGCTCGACCTCT
ACTGCGAACGTGCGCAGATCCAAGATGGTCAGAGTGTTCTGGATCTCGGT
TGCGGTCAAGGCGCGCTGACGCTGCATGTGGCCCAGAAATACAAGAACT
GCCGCGTTACCGCCGTGACCAATAGCGTGAGCCAGAAGGAGTACATCGAA
GAGGAAAGCCGCCGCCGCAATCTGCTGAACGTTGAGGTGAAACTGGCCG
ACATCACCACCCATGAAATGGCGGAAACCTACGACCGCATTCTGGTGATC
GAGCTGTTCGAGCACATGAAGAACTACGAGCTGCTGCTGCGCAAGATCAG
CGAATGGATCAGCAAAGACGGTCTGCTGTTTCTGGAGCACATCTGCCACA
AAACCTTCGCCTACCACTATGAGCCGCTGGATGACGACGACTGGTTCACG
GAGTACGTTTTCCCGGCGGGTACCATGATCATCCCGAGCGCGAGCTTCTT
TCTGTACTTCCAAGATGACGTTAGCGTGGTGAACCATTGGACGCTGAGCG
GCAAGCACTTTAGCCGCACGAACGAAGAGTGGCTGAAACGCCTCGATGC
CAACCTCGACGTTATCAAGCCGATGTTCGAGACCCTCATGGGCAACGAGG
AAGAAGCGGTGAAGCTGATCAACTACTGGCGTGGCTTTTGTCTGAGCGGC
ATGGAGATGTTCGGTTACAACAACGGCGAGGAGTGGATGGCGAGCCATGT
GCTGTTCAAGAAGTAATAACTCGAG

Enzyme expression and purification. *E. coli* strains containing plasmid pWHUIR45, pWHU2491and their variants were inoculated into 500 mL of liquid LB medium (containing the 50 μg mL^{-1} Kanamycin) and grow at 37 °C to an OD_{600} value of 0.6-0.8. After cooled to 18 °C, the cells were induced with 0.1 mM isopropyl-β-Dthiogalactopyranoside (IPTG) for 16 hrs at 18 °C, and then harvested by centrifugation (5000 rpm, 20 min, 4 °C). The cells were resuspended in 20 mL lysis buffer (25 mM HEPES pH 7.5, 300 mM NaCl, 5 mM

imidazole, 10 % glycerol) and lysed by sonication. After centrifugation, 2 mL Ni-NTA agarose resin was added to the supernatant (2 mL L^{-1} of culture) and the solutions were shaken at 4 °C for 1 hr. The protein resin mixtures were loaded onto a gravity flow column, and proteins were eluted with increasing concentrations of imidazole (25 mM, 50 mM, 100 mM and 300 mM) in Buffer A (25 mM HEPES, pH 7.5, 300 mM NaCl, 10% glycerol). Purified proteins were then loaded into PD-10 desalting columns and desalted using buffer B (25 mM HEPES, pH 7.5, 50 mM NaCl, 10% glycerol) and concentrated by centrifugation using an Amicon Ultra-4 (10 KDa, GE Healthcare). Proteins' purity were evaluated by 12 % acrylamide SDS-PAGE and concentration were determined by using a Nanodrop 2000 Spectrophotometer (Thermo Scientific) based on the absorbance of 280 nM. The purified protein was stored at -80°C and used for SDS-PAGE analysis.

1.3. Synthesis of Substrates

General procedure for synthesis of 1a-5a

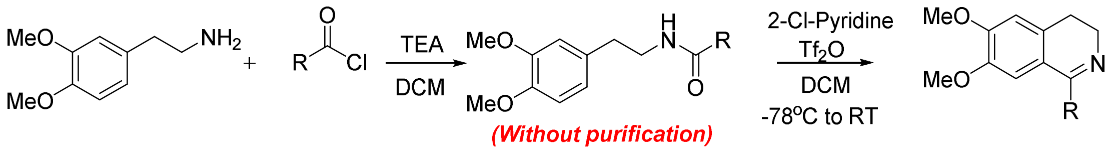

R=3',4'-(CH_2O_2)-Ph (**1a**)
3',4'-$(MeO)_2$-Ph (**2a**)
3',4',5'-$(MeO)_2$-Ph (**3a**)
CH_2Ph (**4a**)
3',4'-$(MeO)_2$-CH_2Ph (**5a**)

1a-5a were prepared according to literature method[3] with slightly modification. 3,4-dimethoxyphenylethylamine (50 mmol, 1.0 equiv) was added in dichloromethane (60 mL) under argon and cool to 0 °C. Triethylamine (TEA, 75 mmol, 1.5 equiv) and arylcarboxyl chloride (50 mmol, 1.0 equiv) were slowly added to the solution. The reaction mixture was stirred at room temperature for 18 hrs and then concentrated under vacuum. The residue was dissolved in EtOAc (40 mL) and washed with 1 M HCl solution (40 mL). The aqueous layer was further extracted with EtOAc (2 × 40 mL). The combined organic extracts were washed with brine (80 mL) and dried over by $MgSO_4$. Solvent was evaporated to give the corresponding amide, which was used in the next step without further purification.

The resulting amide (5 mmol, 1 equiv) was then dissolved into a DCM (30 mL) solution of 2-chloropyridine (6.0 mmol, 1.2 equiv), which was added by trifluoromethanesulfonic anhydride (5.5 mmol, 1.1 equiv) via syringe drop and kept at -78 °C to initiate the reaction. Reaction system was stirred at -78 °C for 15 min and slowly warm to 30 °C overnight. The reaction was quenched with saturated $NaHCO_3$ solution and extracted with ethyl acetate (3 × 50 mL). The combined organic layers were washed with brine, dried over $MgSO_4$, and concentrated under vacuum. The crude product was purified with column chromatography (dichloromethane / methanol) to yield desired product.

Compound **1a**, yield 1.35 g (90%), yellow solid, ^{1}H-NMR (400 MHz, Dimethyl sulfoxide-d6) δ 7.13 (s, 1H), 7.06 (d, 1H), 7.00 (s, 1H), 6.97 (s, 1H), 6.77 (s,1H), 6.10 (s, 2H), 3.84 (s, 3H), 3.63 (s, 3H), 2.63 (t, 2H), 2.00 (t, 2H). For ^{1}H-NMR spectrum see Fig. S11A which is consistent to the literature reported.[4]

Compound **2a**, yield 1.45 g (90%), white solid, ^{1}H-NMR (400 MHz, Chloroform-d) δ 7.24 (d, 1H), 7.16 (d, J=4Hz, 1H), 6.93 (m, 2H), 6.88 (s, 1H), 6.80 (s, 1H) ,3.96 (m, 9H), 3.78-3.80 (m, 2H), 3.76 (s, 3H), 2.71-2.75 (m, 2H). For ^{1}H-NMR spectrum see Fig. S11B which is consistent to the literature reported.[3]

Compound **3a**, yield 1.62 g (91%), white solid, ^{1}H-NMR (400 MHz, Chloroform-d) δ 6.88 (s, 1H), 6.86 (s, 2H), 6.81 (s, 1H), 3.97 (s, 3H), 3.90 (s, 3H), 3.88 (s, 6H), 3.82-3.80 (m, 2H), 3.77 (s, 3H), 2.72-2.76 (m, 2H). For ^{1}H-NMR spectrum see Fig. S11C which is consistent to the literature reported.[3]

Compound **4a**, yield 1.33 g (95%), yellow solid, ^{1}H-NMR (400 MHz, Dimethyl sulfoxide-d6) δ 7.53 (s, 1H), 7.42 (d, 2H), 7.36 (m, 2H), 7.28 (m, 2H), 4.51 (s, 2H), 3.88 (s, 3H), 3.85 (t, 2H), 3.77(s, 3H), 3.02 (t, 2H). For ^{1}H-NMR spectrum see Fig. S11D which is consistent to the literature reported.[5]

Compound **5a**, yield 1.58 g (93%), yellow solid, ^{1}H-NMR (400 MHz, Dimethyl sulfoxide-d6) δ 7.57 (s, 1H), 7.12 (s, 1H), 7.08 (s, 1H), 6.91 (s, 2H), 4.42 (s,2H), 3.88 (s, 3H), 3.82 (t, 2H), 3.80 (s, 3H), 3.72 (t, 6H), 3.00 (t, 2H). For ^{1}H-NMR spectrum see Fig. S11E which is consistent to the literature reported.[6]

General procedure for synthesis of 1b-5b

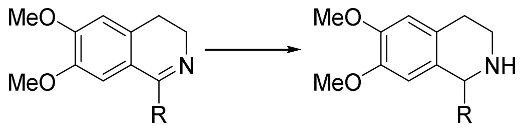

R=3,4-(CH_2O_2)-Ph (**1a**) R=3,4-(CH_2O_2)-Ph (**1b**)
3,4-$(MeO)_2$-Ph (**2a**) 3,4-$(MeO)_2$-Ph (**2b**)
3,4,5-$(MeO)_2$-Ph (**3a**) 3,4,5-$(MeO)_2$-Ph (**3b**)
CH_2Ph (**4a**) CH_2Ph (**4b**)
3,4-$(MeO)_2$-CH_2Ph (**5a**) 3,4-$(MeO)_2$-CH_2Ph (**5b**)

1b-5b were prepared according to the previous method.[2] Sodium borohydride (2 mmol, 2.0 equiv) was first added into a solution of imine products (1 mmol, 1.0 equiv) in methanol (10 mL) at 0 °C under argon. The reaction system was stirred for 2 hrs at room temperature before quenched by 1mol L^{-1} HCl (0.5 mL) and water (20 mL). The mixture was extracted with EtOAC (3 × 50 mL). The organic phases were dried over $MgSO_4$, filtered and concentrated under vacuum to give corresponding amine products as a mixture of diastereoisomers.

Compound **1b**, yield 1.35 g (96 %), yellow solid, $[\alpha]^{23}_D$ - 8.889 (c 0.45, $CHCl_3$), ^{1}H-NMR (400 MHz, Dimethyl sulfoxide-d6) δ 8.82 (d, 1H), 6.73 (d, 1H), 6.67 (d, 2H), 6.21 (s, 1H), 5.97 (s, 2H), 4.83 (s, 1H), 3.71 (s, 3H), 3.50 (s, 3H), 3.44 (t, 2H), 2.79 (m, 2H). For ^{1}H-NMR spectrum see Fig. S11F which is consistent to the literature reported.[7]

Compound **2b**, yield 310.6 mg (95 %), white solid, ^{1}H-NMR (400 MHz, Chloroform-d) δ 6.77-6.83 (m, 3H), 6.64 (s, 1H), 6.27 (s, 1H), 5.00 (s, 1H), 3.88 (s, 6H) ,3.84 (s, 3H), 3.66 (s, 3H), 3.25 (m,1H), 3.06 (m, 1H) ,2.96 (m, 1H), 2.75 (m, 1H). For ^{1}H-NMR spectrum see Fig. S11G which is consistent to the literature reported.[7]

Compound **3b**, yield 332.0 mg (93 %), white solid, ^{1}H-NMR (400 MHz, Chloroform-d) δ 6.65 (s, 1H), 6.50 (s, 2H), 6.32 (s, 1H), 5.00 (s, 1H), 3.90 (s, 3H), 3.86 (s, 3H), 3.82 (s, 6H), 3.69 (s, 3H), 3.27 (m, 1H), 3.08 (m, 1H), 2.96 (m, 1H), 2.77 (m, 1H). For ^{1}H-NMR spectrum see Fig. S11H which is consistent to the literature reported.[3]

Compound **4b**, yield 275 mg (98 %), yellow solid, ^{1}H-NMR (400 MHz, Dimethyl sulfoxide-d6) δ 7.26-7.36 (m, 5H), 6.71 (s, 1H), 6.54 (s, 1H), 4.37 (t, 1H), 3.72 (s, 3H), 3.57 (s, 3H), 3.23 (m, 2H), 3.02 (m, 2H), 2.77 (m, 2H). For ^{1}H-NMR spectrum see Fig. S11I which is consistent to the literature reported.[5]

Compound **5b**, yield 306.9 mg (90 %), white solid, $[\alpha]^{23}_D$ - 2.571 (c 0.35, $CHCl_3$) ,

^{1}H-NMR (400 MHz, Dimethyl sulfoxide-d6) δ 6.96 (s, 1H), 6.94 (d, 1H), 6.78 (s, 1H), 6.64 (s, 1H), 6.54 (s,1H), 4.55 (t,1H), 3.73 (d,9H), 3.62 (s,3H), 3.15 (m,2H), 2.97 (m,2H), 2.89 (m,2H). For ^{1}H-NMR spectrum see Fig. S11J which is consistent to the literature reported.[6]

General procedure for synthesis of 1-5

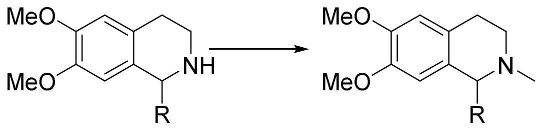

R=3,4-(CH_2O_2)-Ph (**1b**)	R=3,4-(CH_2O_2)-Ph (**1**)
3,4-($(MeO)_2$)-Ph (**2b**)	3,4-($(MeO)_2$)-Ph (**2**)
3,4,5-($(MeO)_2$)-Ph (**3b**)	3,4,5-($(MeO)_2$)-Ph (**3**)
CH_2Ph (**4b**)	CH_2Ph (**4**)
3,4-($(MeO)_2$)-CH_2Ph (**5b**)	3,4-($(MeO)_2$)-CH_2Ph (**5**)

1-5 were prepared according to literature method.[4] The compounds **1b-5b** (1.6 mmol), formaldehyde (35 %, 26 mmol), and formic acid (98 %, 85 mmol) were stirred at reflux under argon atmosphere until the starting material disappeared (2 - 4 h). 10 % $NaHCO_3$ solution was added and the mixture was extracted with CH_2Cl_2 (3 × 50 mL). The organic phases were dried over $MgSO_4$, filtered and concentrated under vacuum to give corresponding products.

Compound **1**, yield 9.5 mg (87 %), white solid, ^{1}H-NMR (400 MHz, Dimethyl sulfoxide-d6) δ 6.87 (s, 1H), 6.85 (s, 1H), 6.69 (s, 2H), 6.12 (s, 1H), 5.99 (d, J = 4.3 Hz, 2H), 4.11 (s, 1H), 3.72 (s, 3H), 3.47 (s, 3H), 2.98 (s, 2H), 2.71 (d, J = 7.2 Hz, 2H), 2.13 (s, 3H). For ^{1}H-NMR spectrum see Fig. S11K which is consistent to the literature reported.[8]

Compound **2**, yield 12.2 mg (86 %), white solid, ^{1}H-NMR (400 MHz, Dimethyl sulfoxide-d6) δ 6.88 (s, 1H), 6.79 (s, 2H), 6.68 (s, 1H), 6.13 (s, 1H), 4.09 (s, 1H), 3.74 (s, 3H), 3.71 (s, 3H), 3.67 (s, 3H), 3.45 (s, 3H), 3.17 (d, J = 5.2 Hz, 1H), 3.01 (d, J = 7.1 Hz, 2H), 2.47 (d, J = 4.1 Hz, 1H), 2.33 (s, 1H), 2.11 (s, 3H). For ^{1}H-NMR spectrum see Fig. S11L which is consistent to the literature reported.[8]

Compound **3**, yield 14.8 mg (89 %), white solid, ^{1}H-NMR (400 MHz, CH_3OH-d) δ 6.73 (s, 1H), 6.60 (s, 2H), 6.19 (s, 1H), 4.28 (s, 1H), 3.81 (d, J = 3.7 Hz, 9H), 3.78 (s, 3H), 3.55 (s, 3H), 2.79 (s, 2H), 2.67 (d, J = 4.1 Hz, 2H), 2.28 (s, 3H). For ^{1}H-NMR spectrum see Fig. S11M which is consistent to the literature reported.[8]

Compound **4**, yield 11.2 mg (85 %), yellow liquid, ^{1}H-NMR (400 MHz, Dimethyl

sulfoxide-d6) δ 7.23 - 7.20 (m, 2H), 7.15 (s, 3H), 6.60 (s, 1H), 6.36 (s, 1H), 3.69 (s, 3H), 3.65 (s, 1H), 3.53 (s, 3H), 3.09 - 3.01 (m, 2H), 2.92 - 2.79 (m, 2H), 2.68 (dd, J = 18.7, 11.7, 4.8 Hz, 2H), 2.36 (s, 3H). For ^{1}H-NMR spectrum see Fig. S11N which is consistent to the literature reported.[4]

Compound **5**, yield 14.6 mg (95 %), pale yellow solid, ^{1}H-NMR (400 MHz, Dimethyl sulfoxide-d6) δ 6.79 (d, J = 8.2 Hz, 1H), 6.71 (d, J = 1.8 Hz, 1H), 6.65 - 6.62 (m, 1H), 6.60 (s, 1H), 6.37 (s, 1H), 3.69 (d, J = 2.2 Hz, 6H), 3.65 (s, 3H), 3.55 (s, 3H), 3.00 (s, 2H), 2.79 (s, 2H), 2.45 (s, 1H), 2.37 (s, 3H). For ^{1}H-NMR spectrum see Fig. S11O which is consistent to the literature reported.[8]

1.4. In Vitro Biochemical Assay

Conversion of DHIQs by IRED. Biotransformations were performed with purified IREDs employing glucose dehydrogenase (GDH)-NADP as a cofactor recycling system. A typical 500 μL reaction mixture contained 20 mM D-glucose, 6 μM GDH, 5 mM $NADP^+$,6 μM IRED, 2 mM imine substrates and 5 % (v/v) DMSO and potassium phosphate buffer (100 mM, pH 7.0). Reactions were incubated at 30 °C with shaking at 200 rpm for 24 hrs, quenched by the addition of 30 μL 10 M NaOH and extracted with ethyl acetate (3 × 300 μL). The organic phase was removed by vacuum and dissolved with methanol then analyzed by HPLC (HPLC condition see 1.6 and Table S5). For achieving accurate value, three repeats were performed and analyzed for each conversion.

Conversion of THIQs by CNMT. A reaction mixture contained 3 mM AdoMet, 5μm CNMT, 0.5 mM amine substrate and 5% (v/v) DMSO and potassium phosphate buffer (100 mM, pH 7.0). Reactions were incubated at 30 °C with shaking at 200 rpm for 24 hrs, extracted with ethyl acetate (3 × 300 μL). The organic phase was removed by vacuum and dissolved with methanol then analyzed by HPLC (HPLC condition see 1.6 and Table S5). For achieving accurate value, three repeats were performed and analyzed for each conversion.

1.5. Determination of Kinetic Constants

Kinetic analysis of IRED. Kinetic parameters for substrates were determined using purified enzyme on Perkin Elmer UV/Vis Spectrometer by monitoring the

decrease of NADPH at 340 nm (ε= 6220 L $M^{-1}cm^{-1}$) at 30°C. Substrate **1a**-**5a** (0.01 mM - 2 mM), NADPH 0.1 mM, IRED (4 μM - 30 μM) , 100 mM (pH 7.0) potassium phosphate (KH_2PO4-K_2HPO4) buffer to the total volume for 1 mL, and the reaction took 60 points per minute and plotted as a curve, which was calculated as the enzyme activity. By calculating the specific enzyme activity at different substrate concentrations, the obtained specific enzyme activity data was fitted by the software Origin 9.0 using the Michaelis constant equation to obtain the kinetic constants K_m and V_{max}. The k_{cat} value and the k_{cat} / K_m value are calculated from V_{max}.

Kinetic analysis of CNMT. Kinetic constants of CNMT were measured by HPLC. Substrate **1b**-**5b** (0.01 mM - 2 mM), 10 μM CNMT, 100 mM (pH 7.0) potassium phosphate (KH_2PO_4-K_2HPO_4) buffer to the total volume for 0.5 mL, the reaction was carried out at 30 °C. Aliquots were withdrawn at each 2 min and mixed with equal volume methanol to terminate the reaction. By centrifugation at 12,000 rpm for 10 min. The supernatants were subjected to HPLC analysis. Kinetic parameters were deduced by non-liner regression analysis based on Michaelis-Menten kinetics using the software Origin 9.0. The k_{cat} value and the k_{cat} / K_m value are calculated from V_{max}.

1.6. HPLC Analysis

Non-chiral HPLC analysis. The conversions of substrates **1a**-**5a** to **1b**-**5b** and **1a**-**5a** to **1**-**5** were measured on a reverse-phase Diamonsil C18 column, 4.6 × 250 mm, 5 μm. Gradient: 0-5 min 20% B, 5-15 min 20-100% B, 15-18 min 100% B, 18-28 min 20% B. Flow rate: 0.8 ml/min. Mobile phase A consisted of H_2O + 0.01% TEA, mobile phase B consisted of acetonitrile +0.01% TEA (also see Table S5). The conversion was calculated based on product generation. Standard with different concentrations were prepared to draw the standard curve which was used for products' quantification in the enzymatic reactions.

Chiral HPLC analysis. For the chiral analysis of the products, two methods are employed. Acetylation was employed for deriverization of **1b**，**2b** and **3b**. 1 μmol of the product was dissolved by 100 μL DCM, 2 mL of triethylamine, 10 μL of acetic anhydride was added. The reaction system was kept at 30 °C and 200

rpm for 2 hrs. The solvent was removed by rotary evaporation, and the final product was dissolved in 200 μL of isopropanol and analyzed by chiral column. The rest of products (**4b**, **5b**) were derivatized with dansyl chloride. 1 μmol of the products with 3 μmol dansyl chloride and 1 μmol triethylene diamine were dissolved in 700 μL DCM, the reaction system was kept at room temperature for 1 hr. Subsequent processing methods are the same as above. Columns used include: Daicel CHIRALPAK AS-H 250 mm × 4.6 mm, 5 μm; Daicel CHIRALPAK AD-H 250 mm × 4.6 mm, 5μm; Daicel CHIRALPAK OD-H 250 mm × 4.6 mm, 5μm; Daicel CHIRALPAK IB-H 250 mm × 4.6 mm, 5μm which is varied depend on different products. Products **1b**-**5b** were separated by isocratic elution and detailed HPLC condition see Table S5C.

1.7. Biotransformation with Recombinant *E. coli* Strains

Co-expression of GDH and IREDs. The GDH gene[9] was cloned into the pACYCDuet-1. The resulted plasmid pWHU2492 was transformed into *E. coli* BL21 (DE_3). This strain was further prepared as competent cell. The pWHUIR45-F190L-W191F and pWHUIR45-F190M-W191F plasmids were individually transformed into this competent cell for co-expression of the IREDs and GDH. Each strains was inoculated into 500 mL of liquid LB medium (containing the 50 μg mL^{-1} Kanamycin and 50 μg mL^{-1} chloramphenicol) and grows at 37 °C for 2-3 hrs to an OD_{600} value of 0.6-0.8. After cooled to 30 °C, the cells were induced with 0.02 mM IPTG. At the meantime, 50 mg L^{-1} imine substrates were also added to the fermentation system. The biotransformation reactions were incubated at 30 °C with shaking at 200 rpm for 3 days. The supernatant was collected by centrifugation at high speed and 10 M NaOH was added to adjust the PH to 10, then extracted with ethyl acetate (3 × 300 μL). The organic phase was removed by distillation and dissolved with methanol then analyzed by HPLC. For achieving accurate value, three repeats were performed and analyzed for each conversion.

Co-expression of GDH, IREDs and CNMT. The CNMT genes were amplified by PCR from plasmid pWHU2491 using primers of CNMT-F and CNMT-R. This DNA fragment was treated by *EcoR*I and *Hin*dIII and cloned into the same site

of pWHU2492. The resulting plasmid pWHU2493 was transformed into *E. coli* BL21(DE3), then prepared as competent cell. The plasmid pWHUIR45-F190M-W191F and pWHUIR45-F190L-W191F was transformed into this competent cell for co-expression with CNMT and GDH. The remaining steps were the same as above.

Enzyme-cell biosynthesis of 2a and 3a. The *E. coli* strain containing the plasmids pWHUIR45-F190L-W191F and pWHU2491 was inoculated into 5 mL LB medium (containing the 50 μg mL^{-1} Kanamycin and 50 μg mL^{-1} chloramphenicol) and grows at 37 °C for 2-3 hrs to an OD_{600} value of 0.6-0.8. After cooled to 30 °C, co-expression of F190L-W191F and GDH was induced by 0.02 mM IPTG and kept for 12 hrs. The cells were then harvested by centrifugation and resuspended in 5 mL 100 mM (pH 7.0) potassium phosphate (KH_2PO4-K_2HPO4) buffer for sonication. This crude lysate was further added into the 5 mL broth of *E. coli* containing CNMT whose OD_{600} value has reached 0.6-0.8 (expression condition of CNMT is identical to F190L-W191F+GDH). In the meantime, 0.02 mM IPTG, 20 mM D-glucose, 1.1 ‰ (w/v) $NADP^+$ and DHIQ substrates **2a** or **3a** (final conc. 50mg mL^{-1}) were supplemented into this mixed system for simultaneously initiating the CNMT expression and cascade biotransformation. These reactions were incubated at 30 °C with shaking at 200 rpm for 3 d before quenched and analyzed by HPLC. For achieving accurate value, three repeats were performed and analyzed for each conversion.

2. Supplementary Tables

Table S1. Kinetic Parameters of IR45 and Its Mutants on the Conversion of **6a** (also see Fig. S3A).

Enzyme	K_m (mM)	K_{cat} (s^{-1})	k_{cat} / K_m ($s^{-1}mM^{-1}$)	K_i
IR45	0.167±0.02	0.322±0.13	1.928±0.60	2.818±0.027
W191A	0.001±0.00	0.015±0.00	15.00±1.53[a]	-
W191L	0.027±0.00	0.13±0.00	0.481±0.03	-
W191H	0.73±0.07	0.034±0.00	0.05±0.00	1.788±0.43
W191M	0.025±0.00	0.009±0.00	0.36±0.00	-
W191N	1.16±0.36	0.016±0.00	0.014±0.00	2.85±2.06
W191C	0.73±0.07	0.034±0.00	0.047±0.00	1.788±0.43
W191S	0.109±0.00	0.034±0.00	0.312±0.00	-
W191F	0.025±0.00	0.171±0.01	6.84±0.48	1.547±0.24

[a] W191A has the biggest catalytic efficency (k_{cat} / K_m value), however its extremely low K_m value makes it readily saturated by substrate when the medium-to-high concentration of substrates was employed. Therefore, its catalytic performance of conversion of **6a** is indeed not as good as W191F (see Fig. S2).

Table S2. Kinetic Parameters of CNMT on the Conversion of **1b**-**5b**.

Sub.	K_m (mM)	K_{cat} (s^{-1})	K_{cat}/K_m ($s^{-1}mM^{-1}$)
**1b**	0.806±0.198	0.098±0.031	0.122±0.038
**2b**	0.289±0.042	0.123±0.014	0.426±0.262
**3b**	0.160±0.033	0.041±0.007	0.256±0.031
**4b**	0.378±0.070	0.499±0.081	1.322±0.080
**5b**	0.807±0.208	0.296±0.042	0.367±0.009

Table S3. Bacterial Strains and Plasmids

Strain/plasmid	Description	Source
E. coli		
DH5*α*	Host for general cloning and protein expression	Invitrogen
BL21 (DE_3)	Host for protein expression	Stratagene
**Plasmids**		
pET28a	Protein expression vector in *E. coli*	Novagen
pWHUIR45	pET28a derivative for IR45 expression	[2]
pWHU2491	pET28a derivative for CNMT expression	This study

pWHUIR45-W191F	pET28a derivative for IR45-W191F expression	This study
pWHUIR45-F190M-W191F	pET28a derivative for IR45-F190M-W191F expression	This study
pWHUIR45-F190L-W191F	pET28a derivative for IR45-F190L-W191F expression	This study
pWHU2492	pACYCDuet derivative for GDH expression	This study

Table S4. Primers Used in the Study.

Mutation site	Forward primer (5' to 3')	Reverse Primer (5' to 3')
W191D	ggatttcttcGATaccagcatgagcggtct	tcatgctggtATCgaagaaatccagcatgc
W191T	ggatttcttcACCaccagcatgagcggtct	tcatgctggtGGTgaagaaatccagcatgc
W191V	ggatttcttcGTGaccagcatgagcggtct	tcatgctggtCACgaagaaatccagcatgc
W191N	ggatttcttcAATaccagcatgagcggtct	tcatgctggtATTgaagaaatccagcatgc
W191H	ggatttcttcCATaccagcatgagcggtct	tcatgctggtATGgaagaaatccagcatgc
W191G	ggatttcttcGGCaccagcatgagcggtct	tcatgctggtGCCgaagaaatccagcatgc
W191Q	ggatttcttcCAGaccagcatgagcggtct	tcatgctggtCTGgaagaaatccagcatgc
W191A	ggatttcttcGCCaccagcatgagcggtct	tcatgctggtGGCgaagaaatccagcatgc
W191P	ggatttcttcCCGaccagcatgagcggtct	tcatgctggtCGGgaagaaatccagcatgc
W191Y	ggatttcttcTACaccagcatgagcggtct	tcatgctggtGTAgaagaaatccagcatgc
W191M	ggatttcttcATGaccagcatgagcggtct	tcatgctggtCATgaagaaatccagcatgc
W191R	ggatttcttcCGCaccagcatgagcggtct	tcatgctggtGCGgaagaaatccagcatgc
W191L	ggatttcttcCTGaccagcatgagcggtct	tcatgctggtCAGgaagaaatccagcatgc
W191S	ggatttcttcAGCaccagcatgagcggtct	tcatgctggtGCTgaagaaatccagcatgc
W191I	ggatttcttcATTaccagcatgagcggtct	tcatgctggtAATgaagaaatccagcatgc
W191E	ggatttcttcGAAaccagcatgagcggtct	tcatgctggtTTCgaagaaatccagcatgc
W191F	ggatttcttcTTCaccagcatgagcggtct	tcatgctggtGAAgaagaaatccagcatgc
W191C	ggatttcttcTGCaccagcatgagcggtct	tcatgctggtGCAgaagaaatccagcatgc
W191K	ggatttcttcAAAaccagcatgagcggtct	tcatgctggtTTTgaagaaatccagcatgc
F190D	gctggatttcGATttcaccagcatgagcgg	tgctggtgaaATCgaaatccagcatgctca
F190T	gctggatttcACCttcaccagcatgagcgg	tgctggtgaaGGTgaaatccagcatgctca
F190V	gctggatttcGTGttcaccagcatgagcgg	tgctggtgaaCACgaaatccagcatgctca
F190N	gctggatttcAATttcaccagcatgagcgg	tgctggtgaaATTgaaatccagcatgctca
F190H	gctggatttcCATttcaccagcatgagcgg	tgctggtgaaATGgaaatccagcatgctca
F190G	gctggatttcGGCttcaccagcatgagcgg	tgctggtgaaGCCgaaatccagcatgctca
F190Q	gctggatttcCAGttcaccagcatgagcgg	tgctggtgaaCTGgaaatccagcatgctca

F190A	gctggatttcGCCttcaccagcatgagcgg	tgctggtgaaGGCgaaatccagcatgctca
F190P	gctggatttcCCGttcaccagcatgagcgg	tgctggtgaaCGGgaaatccagcatgctca
F190Y	gctggatttcTACttcaccagcatgagcgg	tgctggtgaaGTAgaaatccagcatgctca
F190M	gctggatttcATGttcaccagcatgagcgg	tgctggtgaaCATgaaatccagcatgctca
F190R	gctggatttcCGCttcaccagcatgagcgg	tgctggtgaaGCGgaaatccagcatgctca
F190L	gctggatttcCTGttcaccagcatgagcgg	tgctggtgaaCAGgaaatccagcatgctca
F190S	gctggatttcAGCttcaccagcatgagcgg	tgctggtgaaGCTgaaatccagcatgctca
F190I	gctggatttcATTttcaccagcatgagcgg	tgctggtgaaAATgaaatccagcatgctca
F190E	gctggatttcGAAttcaccagcatgagcgg	tgctggtgaaTTCgaaatccagcatgctca
F190W	gctggatttcTGGttcaccagcatgagcgg	tgctggtgaaCCAgaaatccagcatgctca
F190C	gctggatttcTGCttcaccagcatgagcgg	tgctggtgaaGCAgaaatccagcatgctca
F190K	gctggatttcAAAttcaccagcatgagcgg	tgctggtgaaTTTgaaatccagcatgctca

Table S5. HPLC Conditions Used for Analysis of the Products.

(A) HPLC conditions used for analysis of **1b**-**5b**.

Sub.	Wavelength [nm]	retention time (min)	
		T_1 [a]	T_2 [b]
**1a**	286	17.4	17.2
**2a**	286	16.8	16.4
**3a**	282	16.7	16.5
**4a**	287	17.2	18.3
**5a**	287	16.3	17

a: retention time of **1a**-**5a**. b: retention time of **1b**-**5b**.

(B) HPLC conditions used for analysis of **1**-**5**.

Sub.	Wavelength [nm]	retention time (min)	
		T_1 [a]	T_2 [b]
**1a**	286	17.4	19.4
**2a**	286	16.8	18
**3a**	282	16.7	18.2
**4a**	287	17.2	20
**5a**	287	16.3	18.2

a: retention time of **1a**-**5a**. b: retention time of **1**-**5**.

(C) HPLC columns and conditions used for the chiral analysis of **1b**-**5b**.

Sub.	Column	Wavelength [nm]	Flow rate [mL min^{-1}]	Solvent [a] A: B	Amine retention time (min) [b]	
					T_1	T_2
**1b**	Chiralpak OD-H	254	1.0	90:10	26.97 (*S*)	42.01 (*R*)
**2b**	Chiralpak AS-H	230	1.0	85:15	41.40 (*S*)	95.98 (*R*)
**3b**	Chiralpak AD-H	210	1.0	80:20	23.48 (*S*)	49.45 (*R*)
**4b**	Chiralpak IB-H	254	0.5	80:20	19.79 (*S*)	25.39 (*R*)
**5b**	Chiralpak OD-H	254	0.8	80:20	24.82 (*S*)	35.58 (*R*)

a: Solvent A: n-hexane; Solvent B: isopropanol

b: *R*- and *S*- annotation was according to the literatures[3,6]

3. Supplementary Figures

Fig. S1. HPLC analysis of imines reduction. Formation of products (**1b**-**5b**) are quantified based on standard curves of **1b-5b**, and their conversions in each reaction are indicated in percentage (in red). The retention time of the precursors **1a**-**5a** are indicated by the dashed lines in blue. Samples of the reaction systems are indicated by the solid lines. (A) Reduction of **2a** and **3a** by W191F+GDH through in vitro enzymatic reactions. (B) Reduction of **1a-5a** by F190M-W191F+GDH and F190L-W191F+GDH through in vitro enzymatic reactions. (C) Reduction of **1a-5a** by the *E. coli* strains with F190M-W191F+GDH or F190L-W191F+GDH.

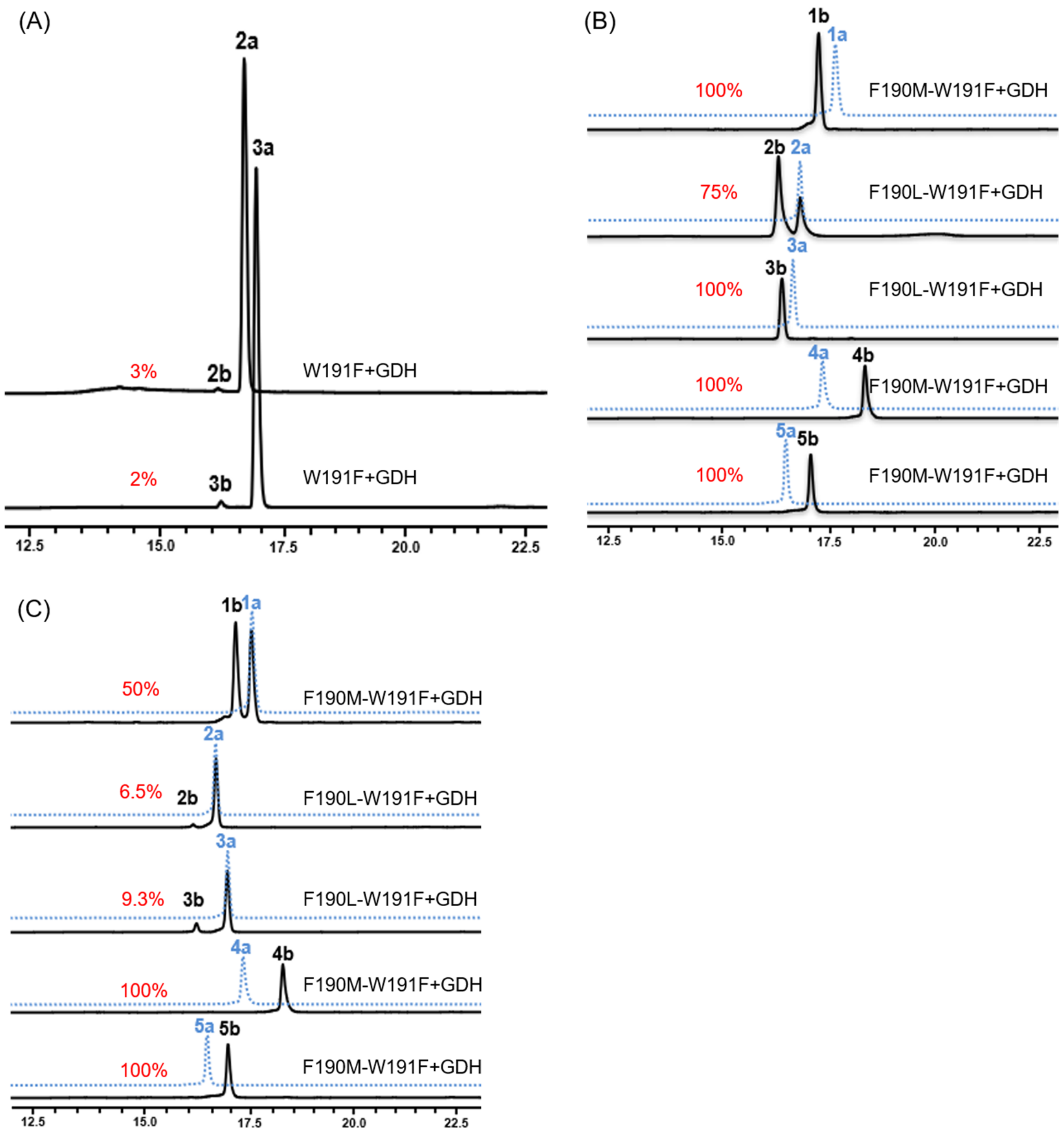

Fig. S2. Enzyme activity of IR45 and its mutants on the conversion of **6a**.

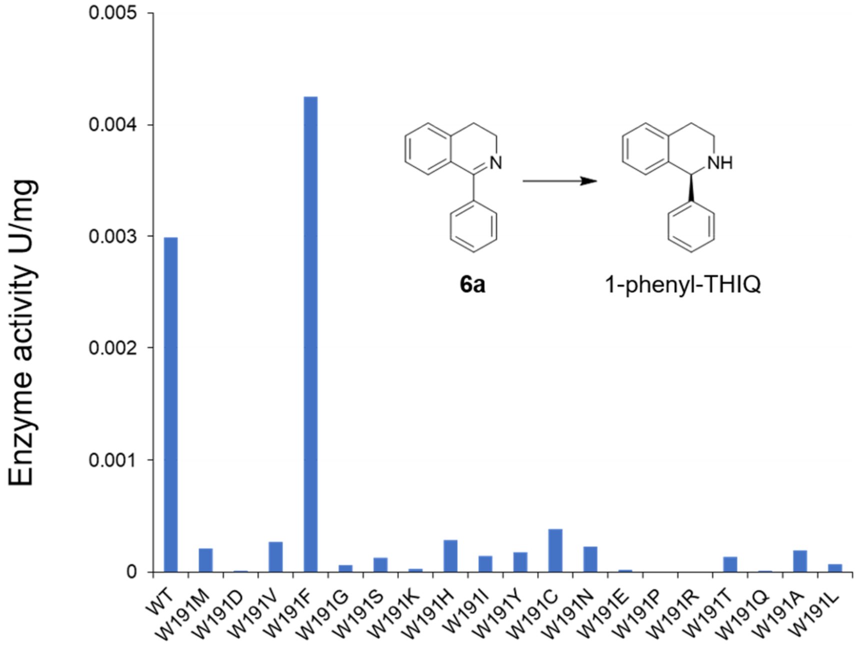

Fig. S3. Kinetic analysis of the IRED and CNMT reactions. (A) Kinetic data of IR45 and its mutants on the conversion of **6a**. (B) Kinetic data of F190L-W191F, F190M-W191F and wild-type IR45 on the conversion of **1a**-**5a**. (C) Kinetic data of CNMT on the conversion of **1b**-**5b**.

(A)

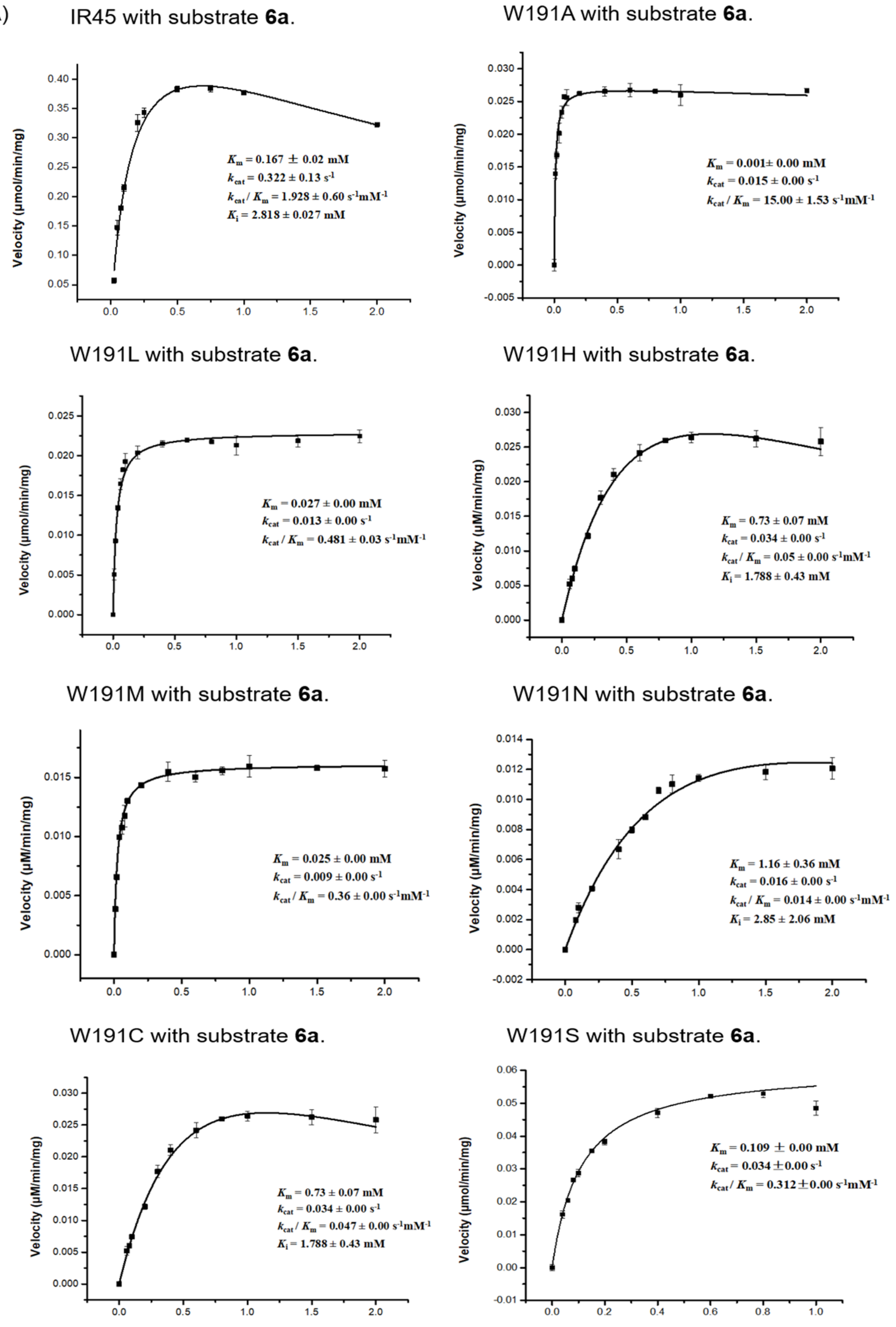

W191F with substrate **6a**.

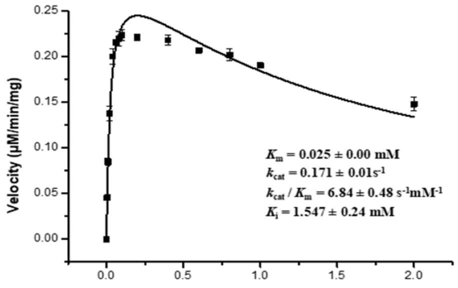

(B) IR45 with substrate **1a**.

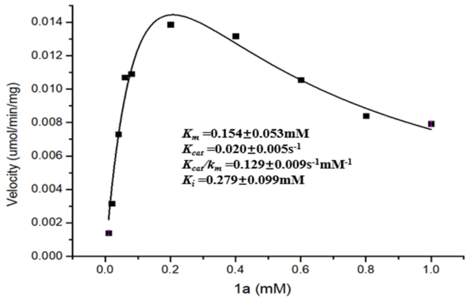

IR45 with substrate **4a**.

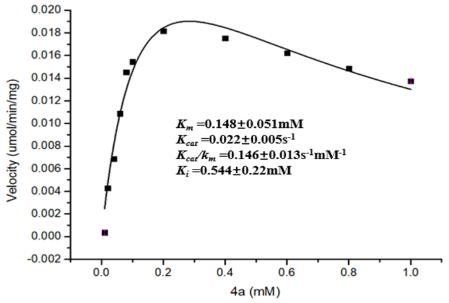

IR45 with substrate **5a**.

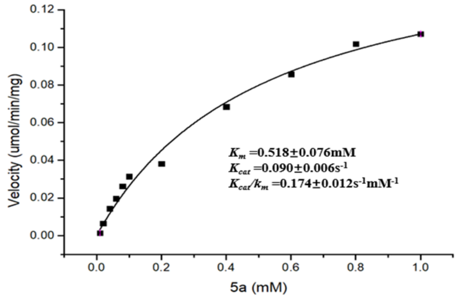

F190L-W191F with substrate **1a**.

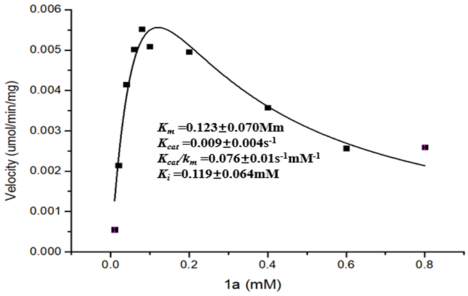

F190L-W191F with substrate **2a**.

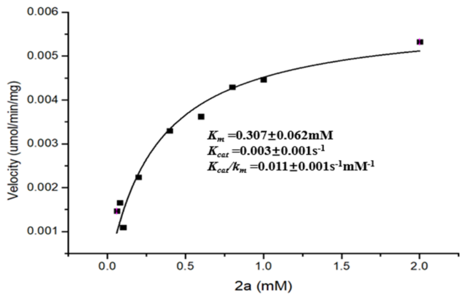

F190L-W191F with substrate **3a**.

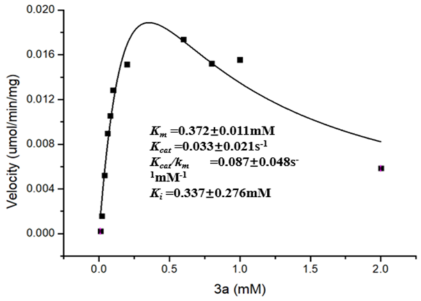

F190L-W191F with substrate **4a**.

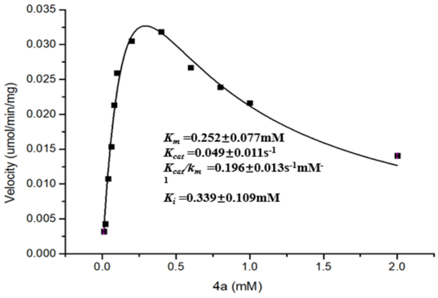

F190L-W191F with substrate **5a**.

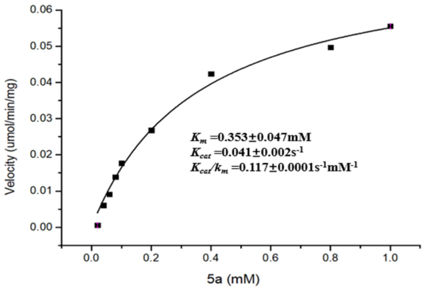

F190M-W191F with substrate **1a**.

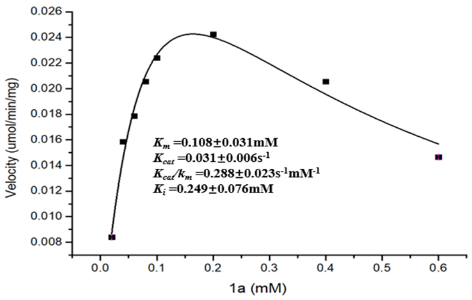

F190M-W191F with substrate **4a**.

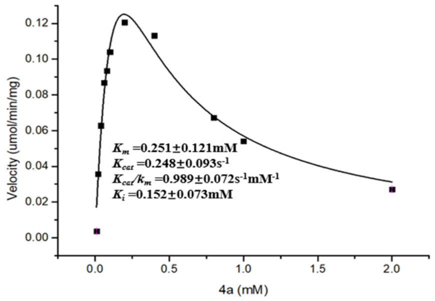

F190M-W191F with substrate **5a**.

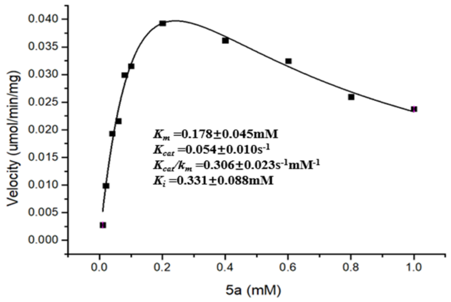

(C) CNMT with substrate **1b**.

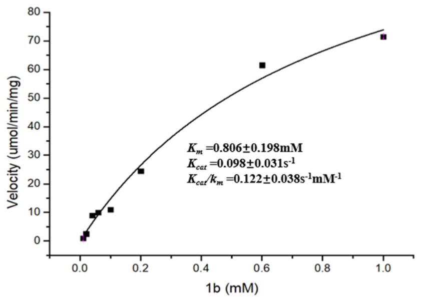

CNMT with substrate **2b**.

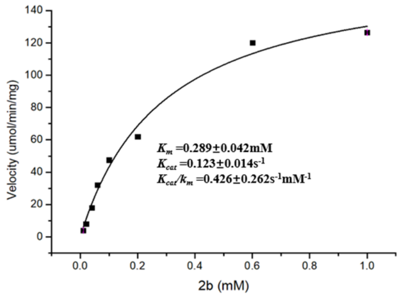

CNMT with substrate **3b**.

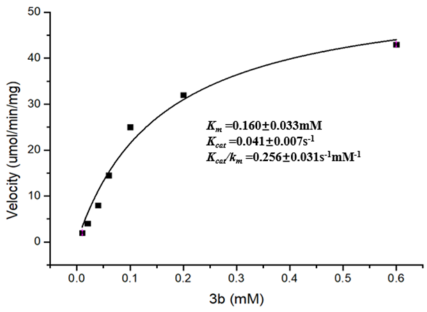

CNMT with substrate **4b**.

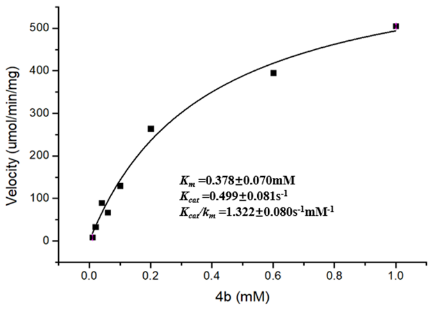

CNMT with substrate **5b**.

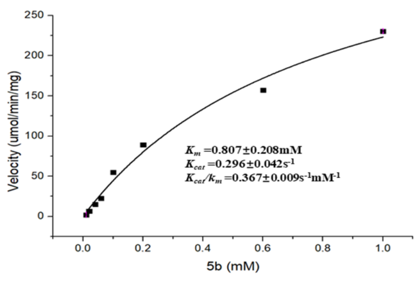

Fig. S4. Reported approach for microbial production of racemic (*R*, *S*)-laudanosine from (*R*, *S*)-norlaudanosoline.[10] Incubation of mixed *E. coli* strains harboring the pGFLOMT1 (O-methyltransferase-1 from the *Glaucium flavum*), pGFLOMT6 (O-methyltransferase-6 from the *Glaucium flavum*), and pCNMT (NMT from the *C. japonica*) with (*R*, *S*)-norlaudanosoline produced five compounds with m/z 330, m/z 330, m/z 344, m/z 344, and m/z 358, which were identified as (*R*, *S*)-reticuline (**17**), (*R*, *S*)-norcodamine (**6**), (*R*, *S*)-codamine (**9**), (*R*, *S*)-laudanine (**10**), and (*R*, *S*)-laudanosine (**13**, target product), respectively.

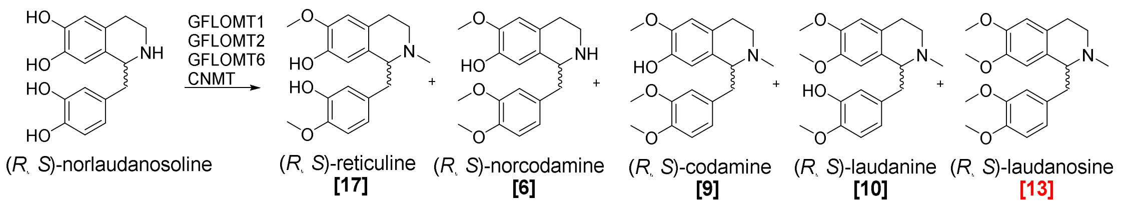

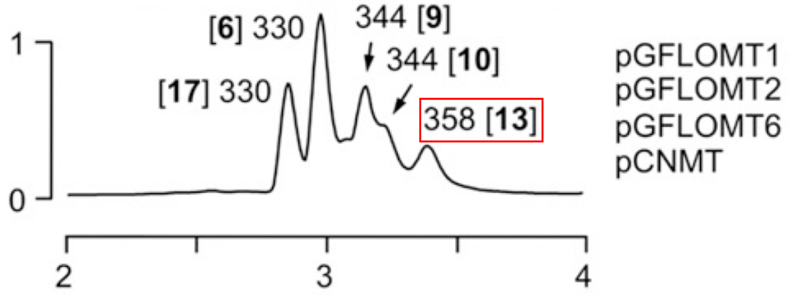

Fig. S5. HPLC analysis of the N-methylation reactions. Conversions in each reaction are indicated in percentage (in red). (A) Methylation of **1b-5b** by CNMT through in vitro enzymatic reactions. (B) Methylation of **1b-5b** by the *E. coli* strain containing CNMT.

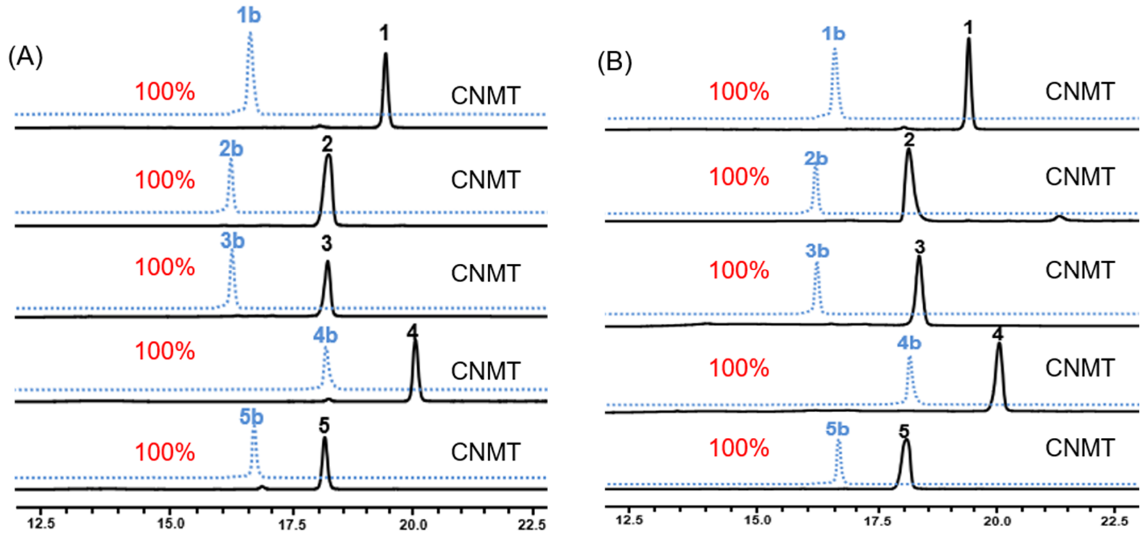

Fig. S6. *Insilico* models of CNMT with the substrate **1** and **5**. (A) The structure of CNMT with adenosylhomocysteine (AdoHcy, in purple) and the product **1** (in cyan), showing key amino acid residues involved in catalysis or substrate recognition. (B) The structure of CNMT with AdoHcy (in purple) and the product **5** (in cyan) showing key amino acid residues involved in catalysis or substrate recognition.

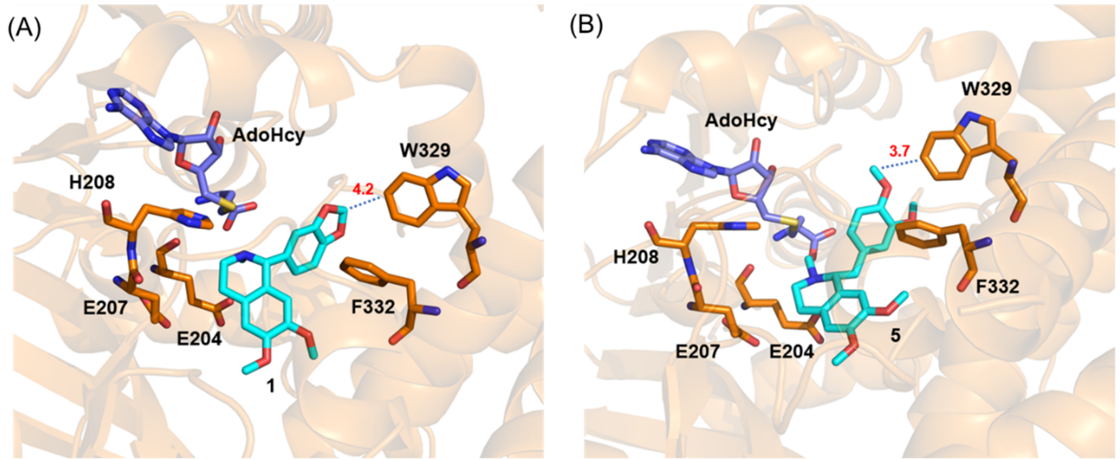

Fig. S7. HPLC analysis of the imine reduction and N-methylation reactions. Conversions in each reaction are indicated in percentage (in red). (A) Conversion of **1a**-**5a** to **1**-**5** by F190M-W191F+GDH+CNMT-W329F and F190L-W191F+GDH+CNMT-W329F through in vitro enzymatic reactions. (B) Conversion of **1a**-**5a** to **1**-**5** by F190M-W191F+GDH+CNMT and F190L-W191F+GDH+CNMT through in vitro enzymatic reactions. (C) Conversion of **1a**-**5a** to **1**-**5** by the *E*. *coli* strains containing F190M-W191F+GDH+CNMT and F190L-W191F+GDH+CNMT.

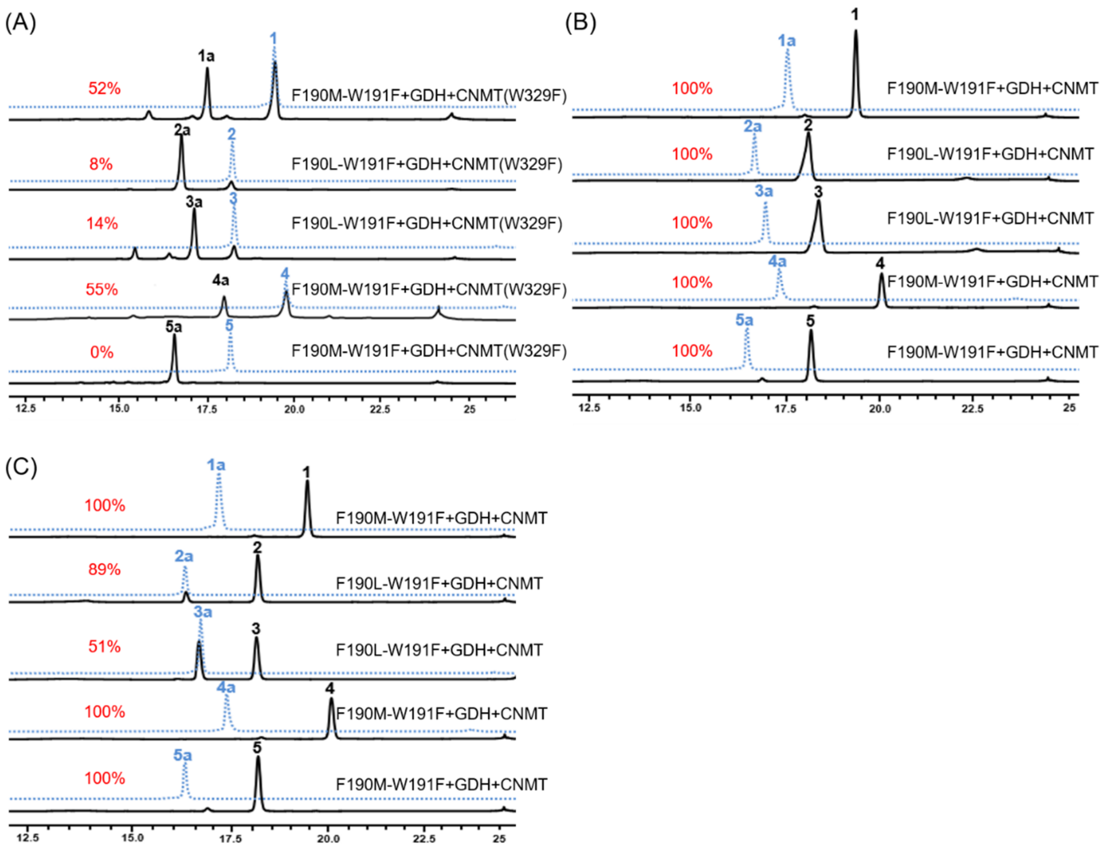

Fig. S8. Effects on the in vivo imine reduction by different IPTG concentration and temperature. (A) Reduction of **1a** by the *E. coli* strain containing F190M-W191F+GDH under 30°C using different concentrations of IPTG. (B) Reduction of **1a** by the *E. coli* strain containing F190M-W191F+GDH at 20 μM IPTG under different culturing temperatures.

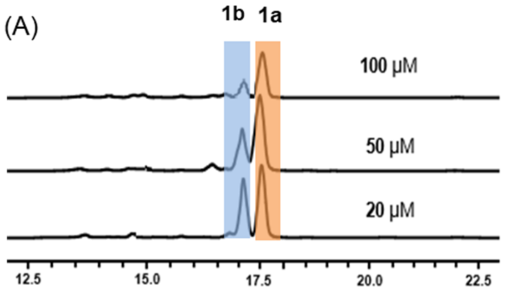

IPTG (μM)	Conv. (%)
100	18
50	36
20	49

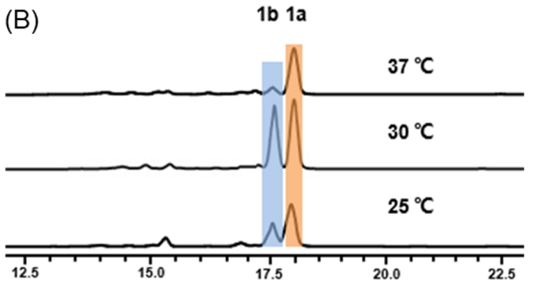

Temperature (℃)	Conv. (%)
37	6
30	49
25	10

Fig. S9. 12% SDS-PAGE analysis of proteins. (A) 12% SDS-PAGE analysis of IR45 and its mutants. Lane M: protein marker. (B) 12% SDS-PAGE analysis of CNMT.

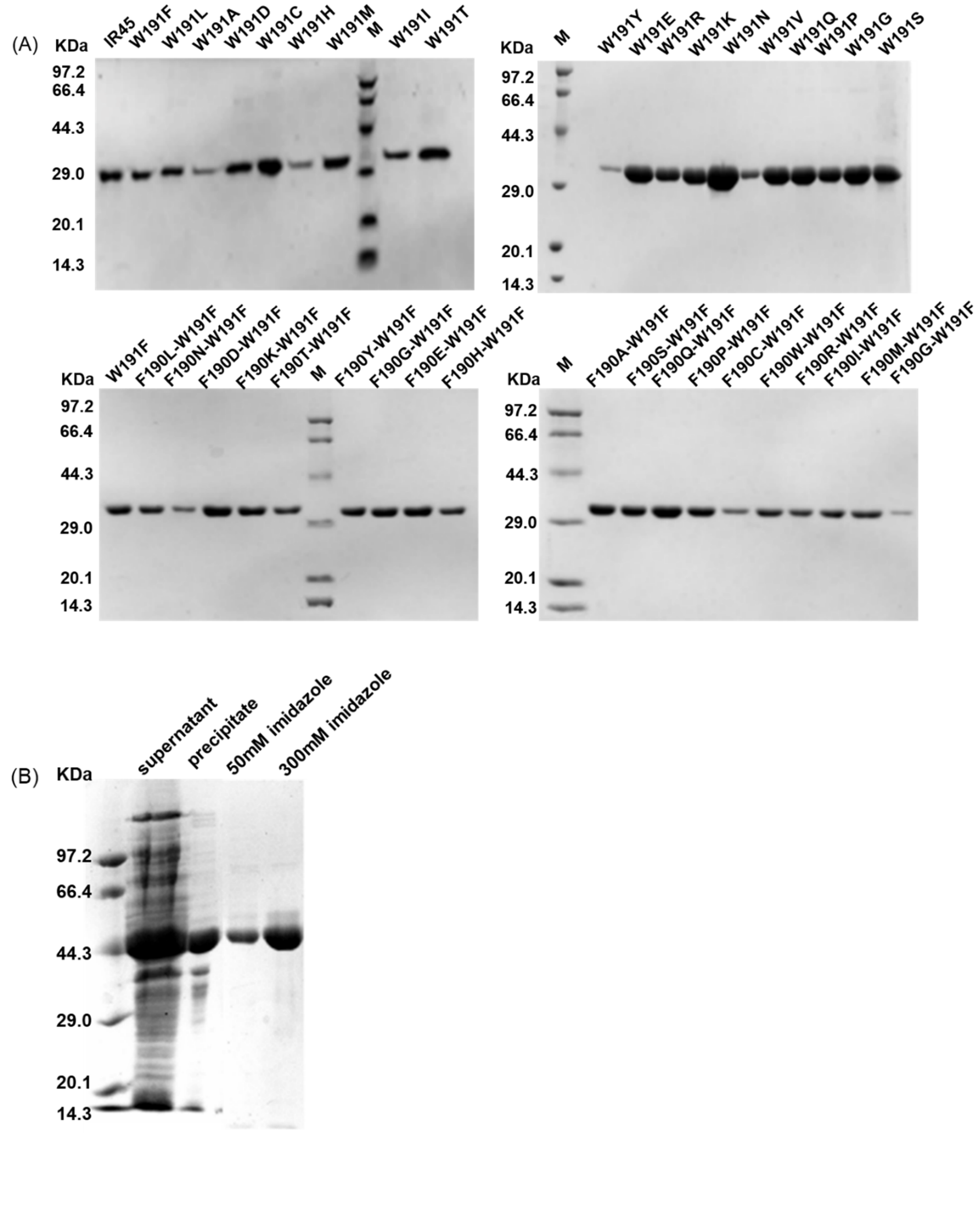

Fig. S10. Chiral HPLC analysis of the enantiomeric purity of the imine products **1b**-**5b**.

(A) Conversion of **1a** into **1b**. Standard racemic amine **1b** (top), followed by the products of wild-type IR45, F190L-W191F and F190M-W191F.

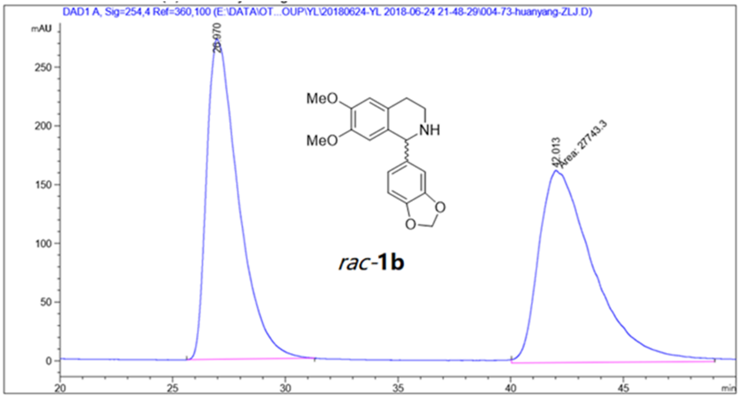

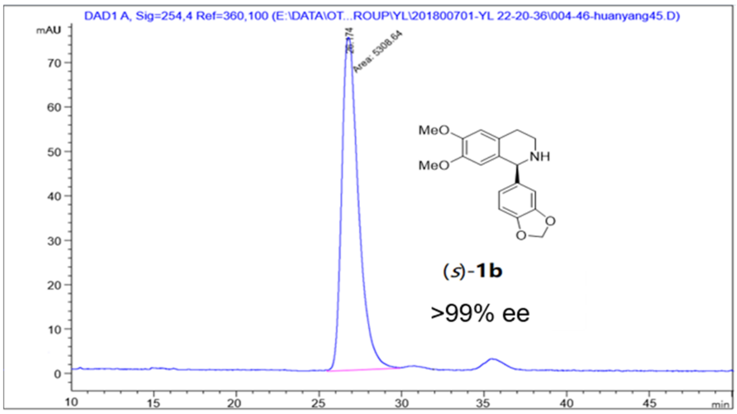

Peak #	RetTime [min]	Type	Width [min]	Area [mAU*s]	Height [mAU]	Area %
1	26.174	MM	1.1809	5308.64404	74.92442	100.0000
Totals :				5308.64404	74.92442	

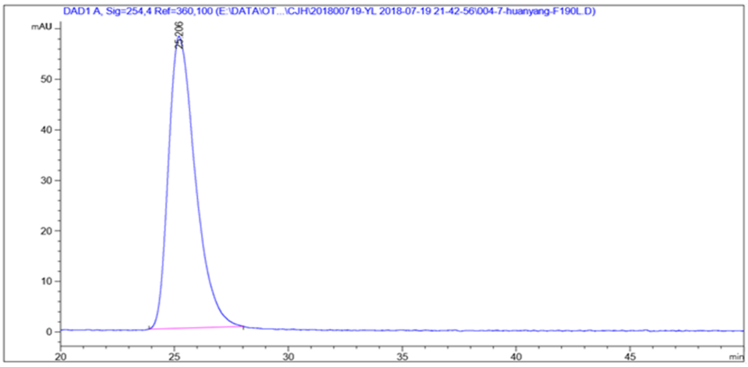

Peak #	RetTime [min]	Type	Width [min]	Area [mAU*s]	Height [mAU]	Area %
1	25.206	BB	0.9560	4734.58301	57.84018	100.0000
Totals :				4734.58301	57.84018	

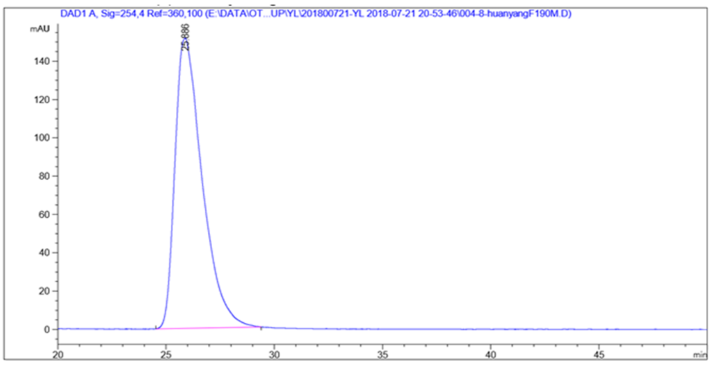

Peak #	RetTime [min]	Type	Width [min]	Area [mAU*s]	Height [mAU]	Area %
1	25.886	BB	1.0229	1.32368e4	151.42238	100.0000
Totals :				1.32368e4	151.42238	

(B) Conversion of **2a** into **2b**. Standard racemic amine **2b** (top), followed by the product of F190L-W191F.

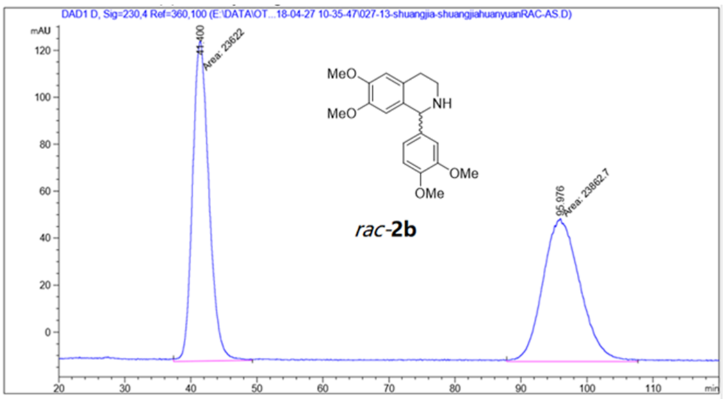

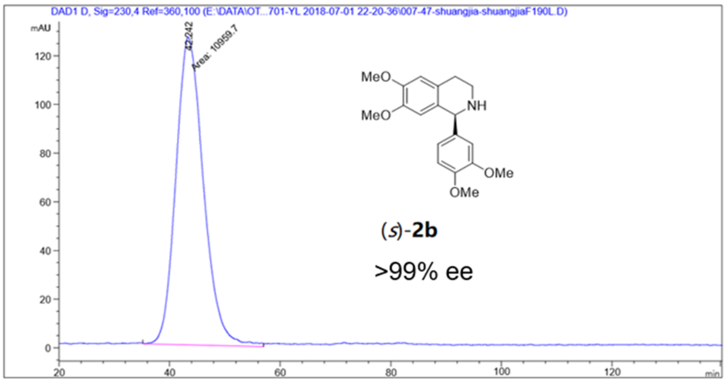

Peak #	RetTime [min]	Type	Width [min]	Area [mAU*s]	Height [mAU]	Area %
1	42.242	MM	1.4444	1.09597e4	126.46484	100.0000
Totals :				1.09597e4	126.46484	

(C) Conversion of **3a** into **3b**. Standard racemic amine **3b** (top), followed by the product of F190L-W191F.

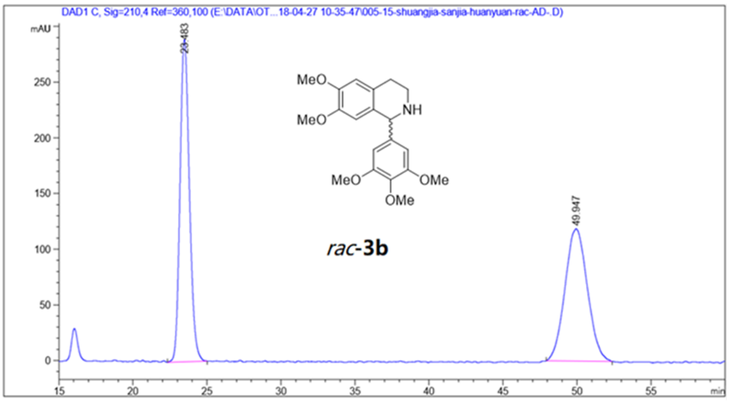

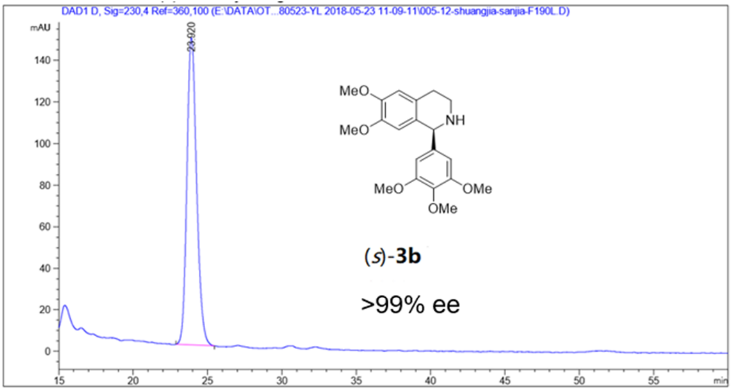

Peak #	RetTime [min]	Type	Width [min]	Area [mAU*s]	Height [mAU]	Area %
1	23.920	BB	0.6412	6712.15771	147.68477	100.0000
Totals :				6712.15771	147.68477	

(D) Conversion of **4a** into **4b**. Standard racemic amine **4b** (top), followed by the products of wild-type IR45, F190L-W191F and F190M-W191F.

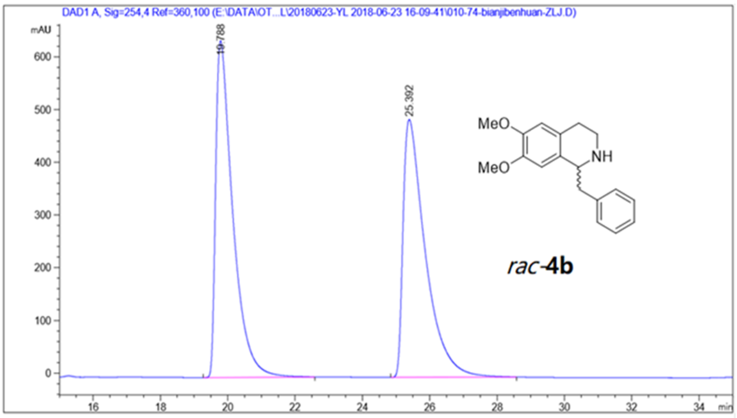

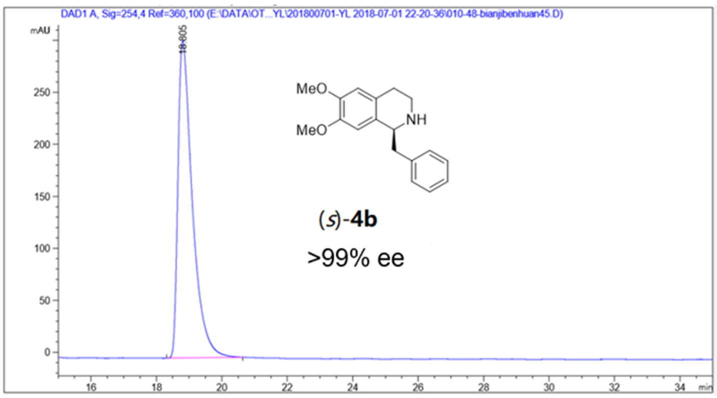

Peak #	RetTime [min]	Type	Width [min]	Area [mAU*s]	Height [mAU]	Area %
1	18.805	BB	0.4269	9324.95801	305.21124	100.0000
Totals :				9324.95801	305.21124	

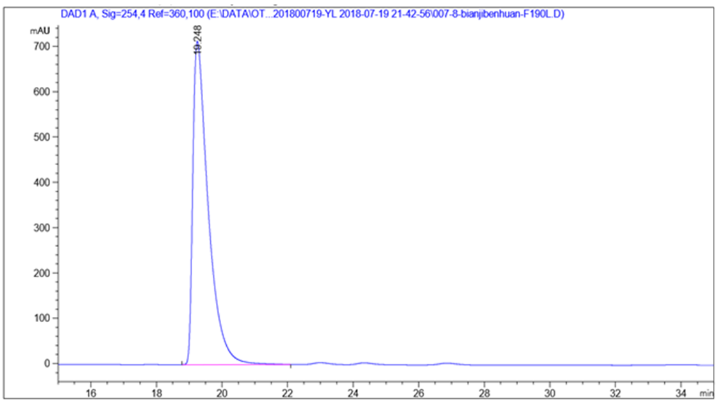

Peak #	RetTime [min]	Type	Width [min]	Area [mAU*s]	Height [mAU]	Area %
1	19.248	BB	0.4601	2.33147e4	713.72571	100.0000
Totals :				2.33147e4	713.72571	

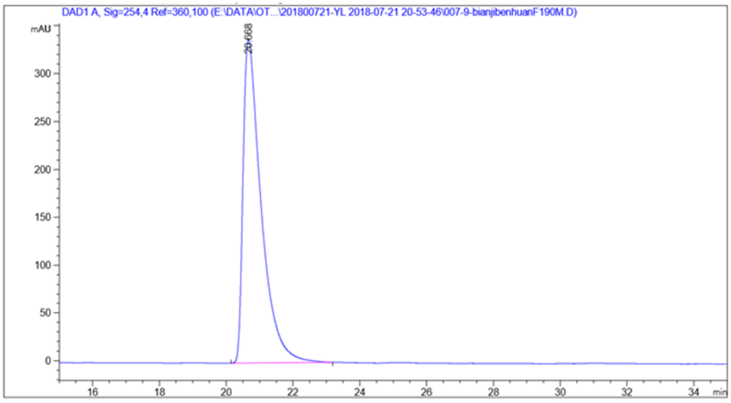

Peak #	RetTime [min]	Type	Width [min]	Area [mAU*s]	Height [mAU]	Area %
1	20.668	BB	0.5277	1.27367e4	337.95218	100.0000
Totals :				1.27367e4	337.95218	

(E) Conversion of **5a** into **5b**. Standard racemic amine **5b** (top), followed by the products of wild-type IR45, F190L-W191F and F190M-W191F.

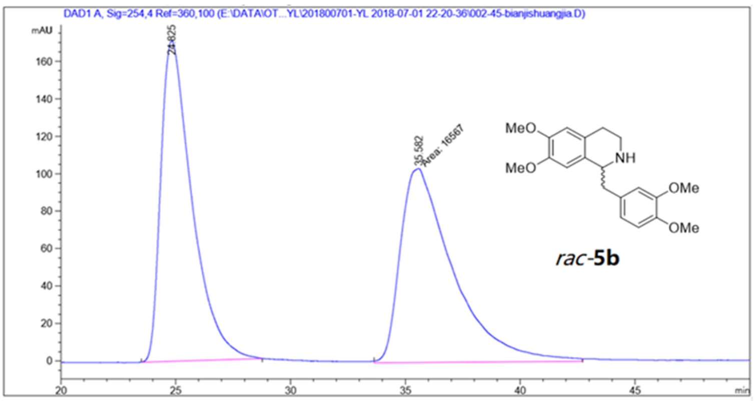

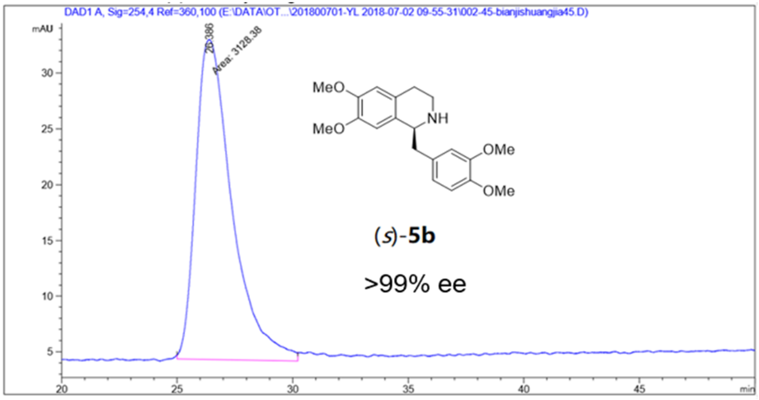

Peak #	RetTime [min]	Type	Width [min]	Area [mAU*s]	Height [mAU]	Area %
1	26.386	MM	1.8159	3128.38257	28.71299	100.0000
Totals :				3128.38257	28.71299	

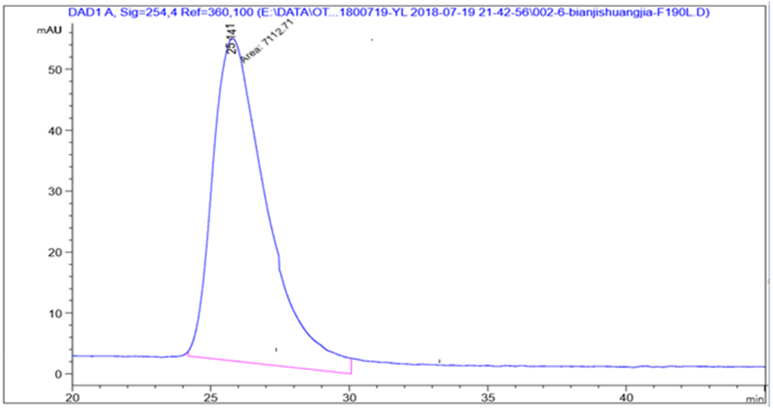

Peak #	RetTime [min]	Type	Width [min]	Area [mAU*s]	Height [mAU]	Area %
1	25.141	MM	2.2443	7112.70947	52.82122	100.0000
Totals :				7112.70947	52.82122	

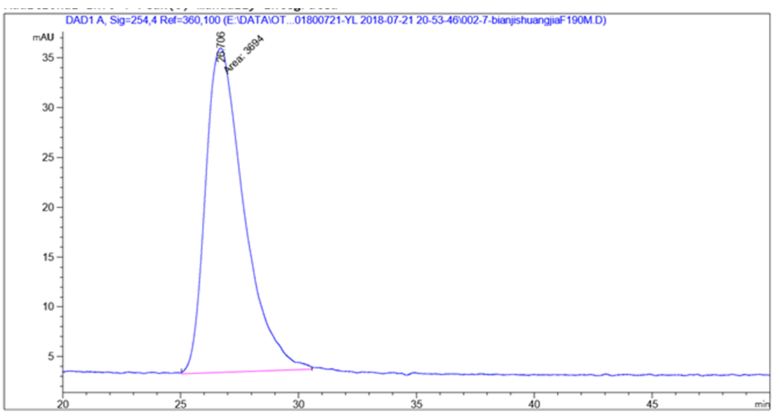

Peak #	RetTime [min]	Type	Width [min]	Area [mAU*s]	Height [mAU]	Area %
1	26.706	MM	1.8898	3694.00366	32.57784	100.0000

Totals : 3694.00366 32.57784

Fig. S11. NMR spectrum of synthetic compounds.

(A) ^{1}H NMR spectra of the compound **1a** recorded in DMSO.

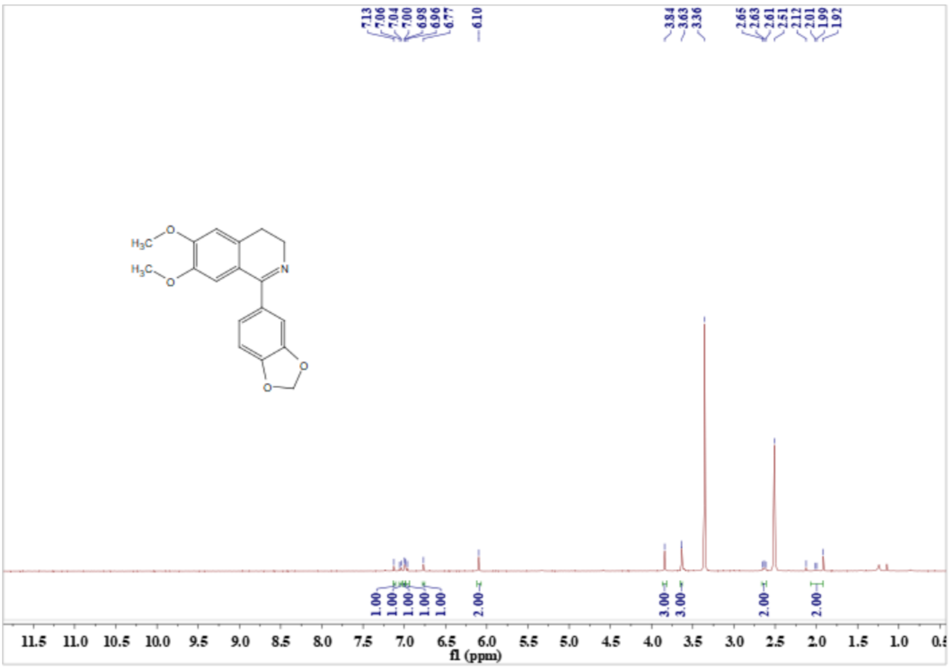

(B) ^{1}H NMR spectra of the compound **2a** recorded in $CDCl_3$.

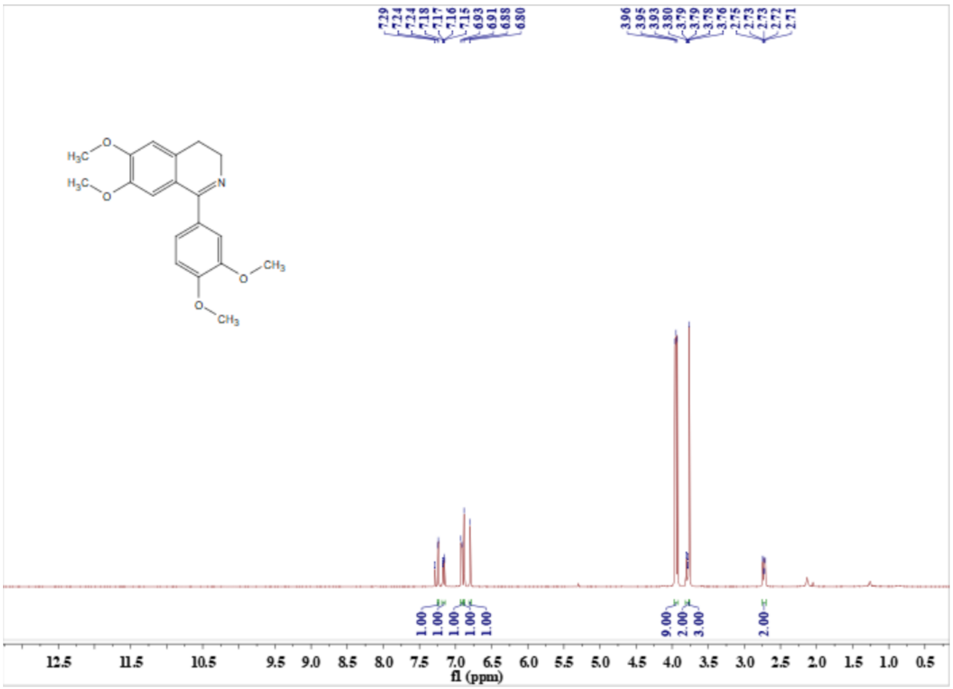

(C) ^{1}H NMR spectra of the compound **3a** recorded in $CDCl_3$.

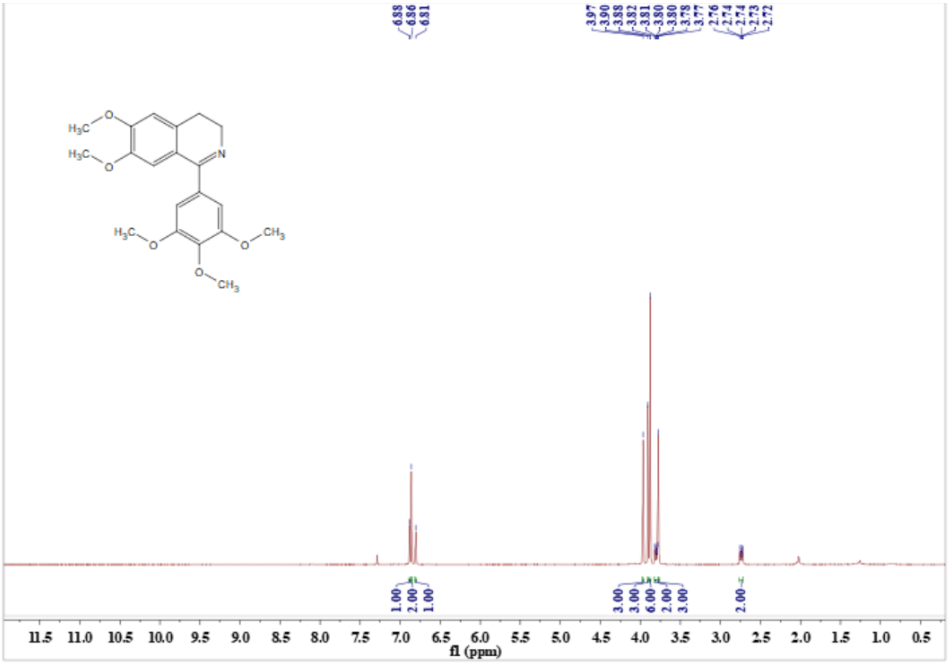

(D) ^{1}H NMR spectra of the compound **4a** recorded in DMSO.

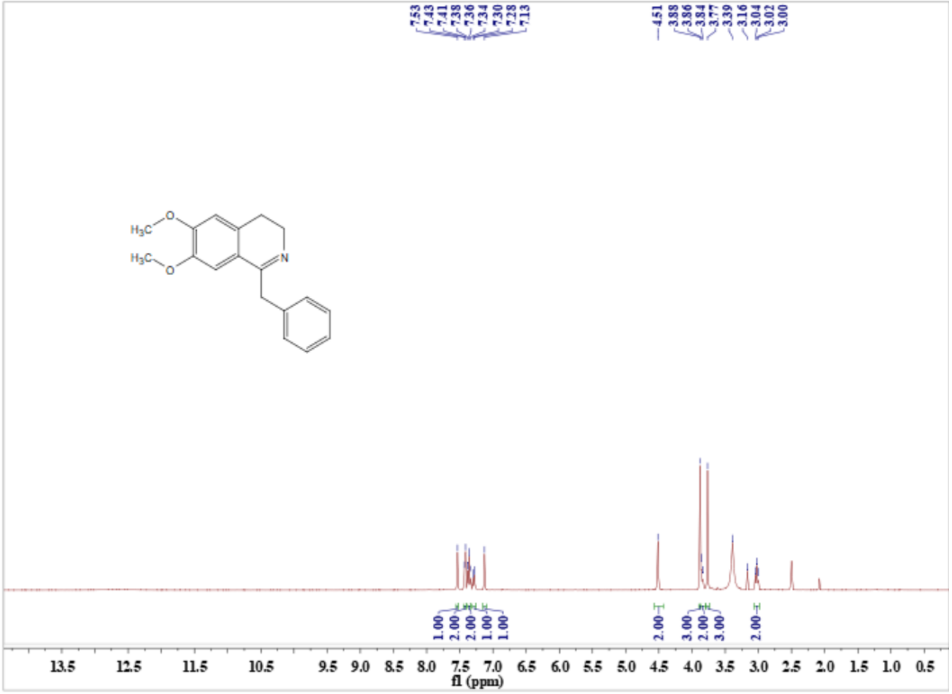

(E) ^{1}H NMR spectra of the compound **5a** recorded in DMSO.

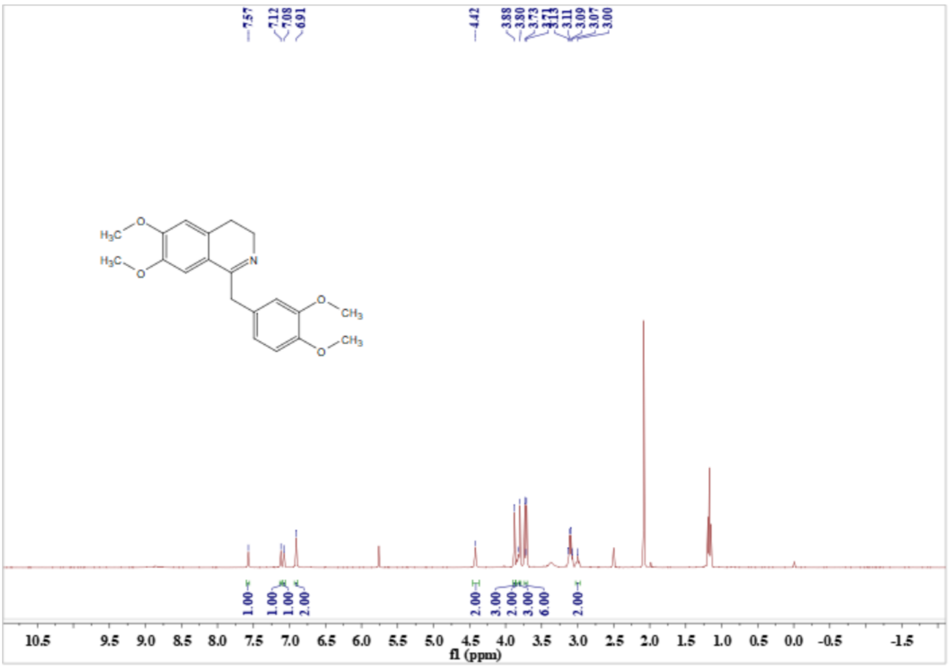

(F) ^{1}H NMR spectra of the compound **1b** recorded in DMSO.

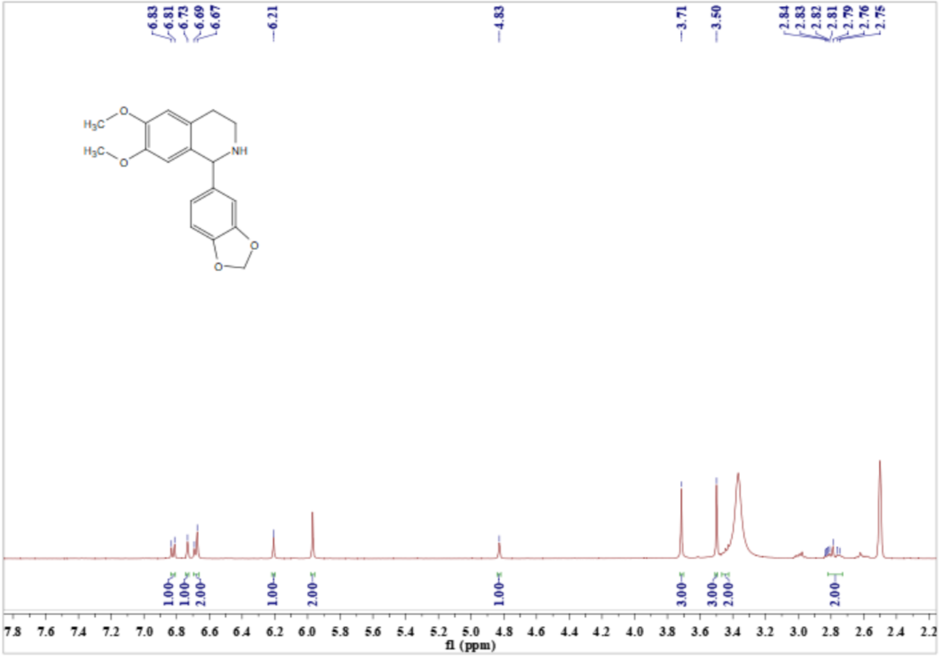

(G)^{1}H NMR spectra of the compound **2b** recorded in $CDCl_3$.

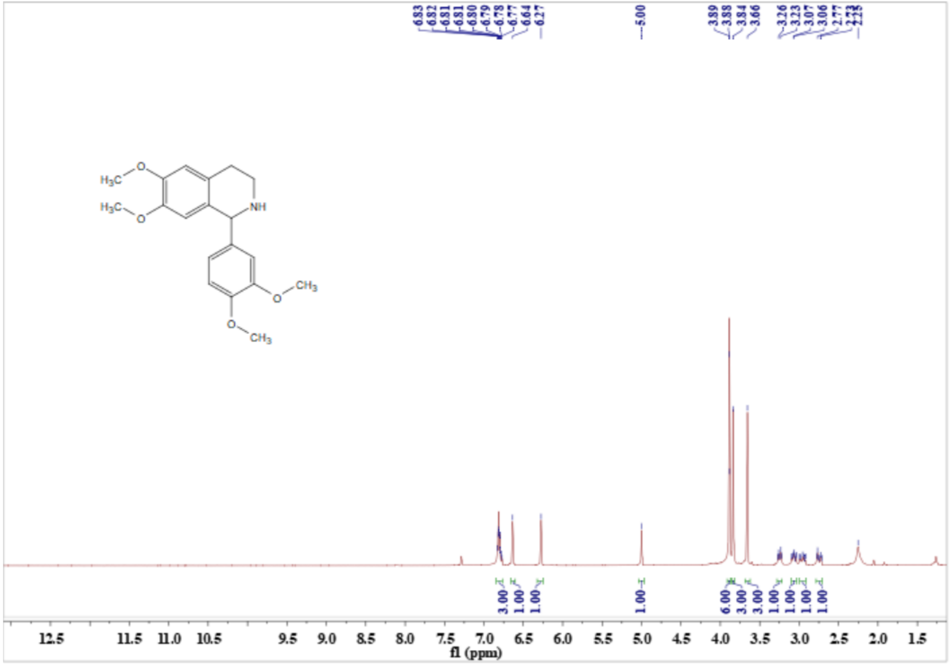

(H)^{1}H NMR spectra of the compound **3b** recorded in $CDCl_3$.

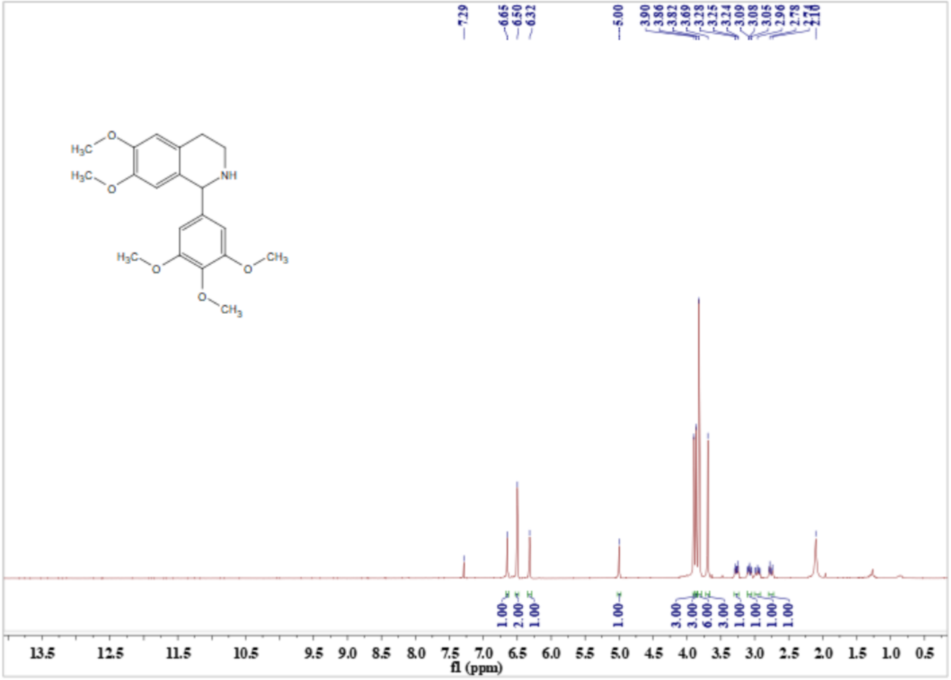

(I) ^{1}H NMR spectra of the compound **4b** recorded in DMSO.

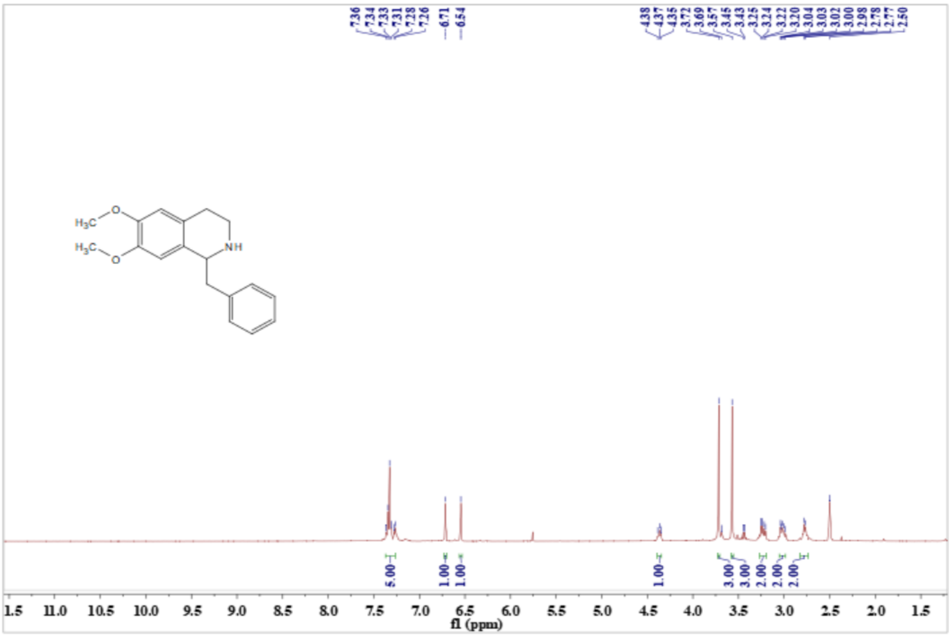

(J) ^{1}H NMR spectra of the compound **5b** recorded in DMSO.

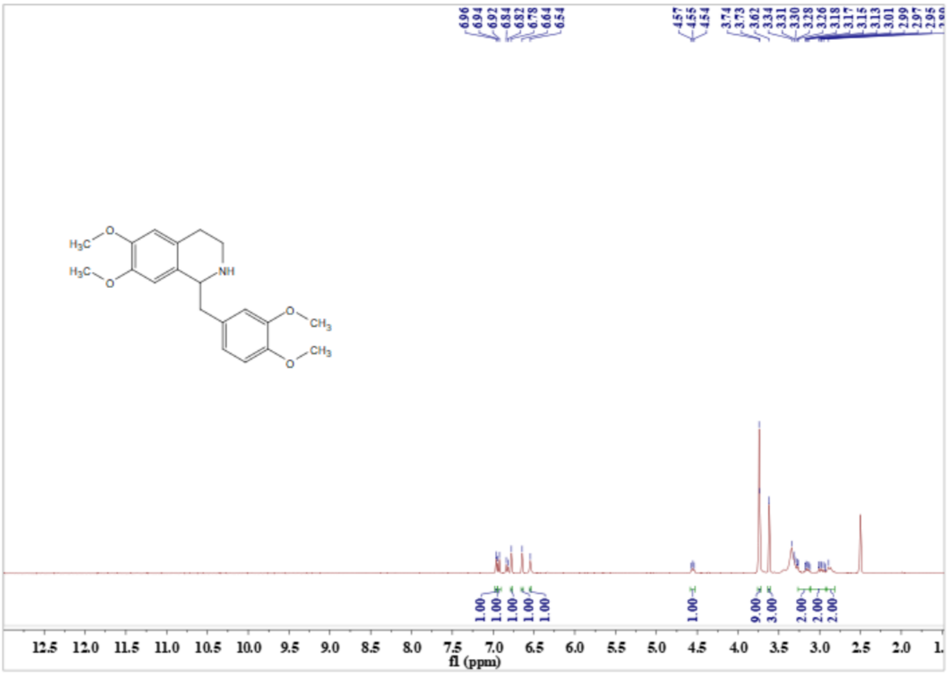

(K) ^{1}H NMR spectra of the compound **1** recorded in DMSO.

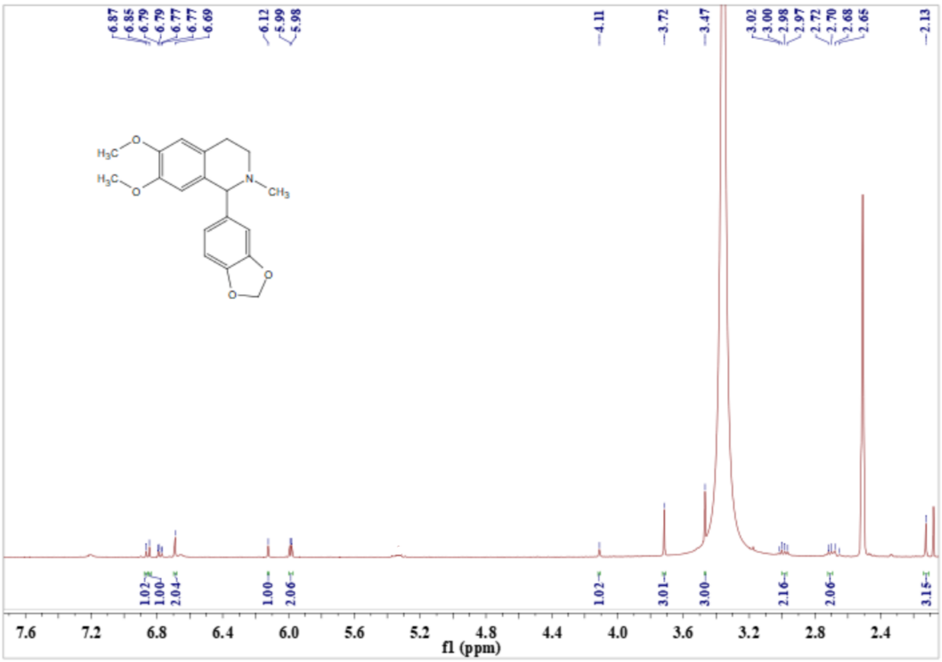

(L) ^{1}H NMR spectra of the compound **2** recorded in DMSO.

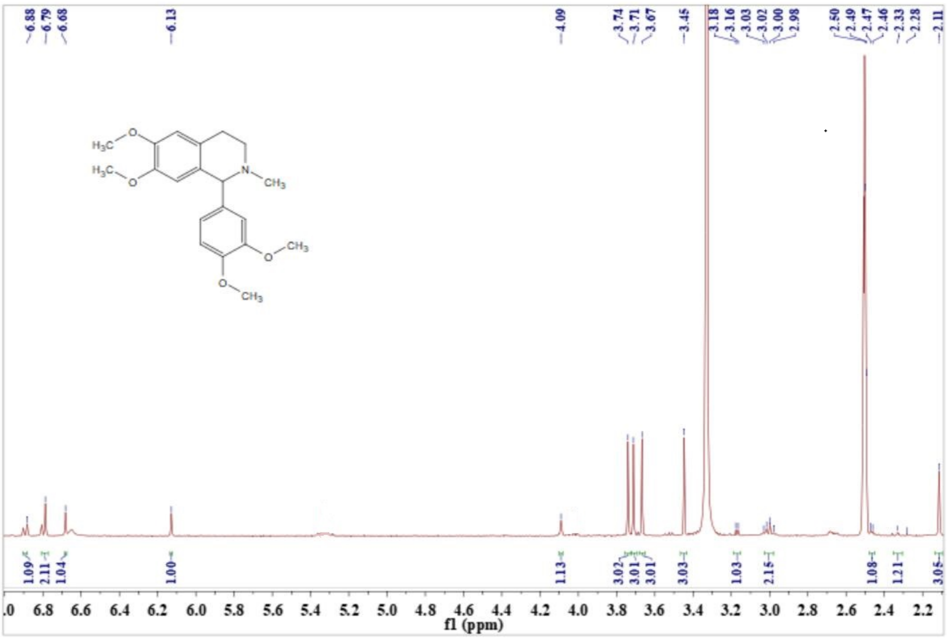

(M)^{1}H NMR spectra of the compound **3** recorded in MeOH.

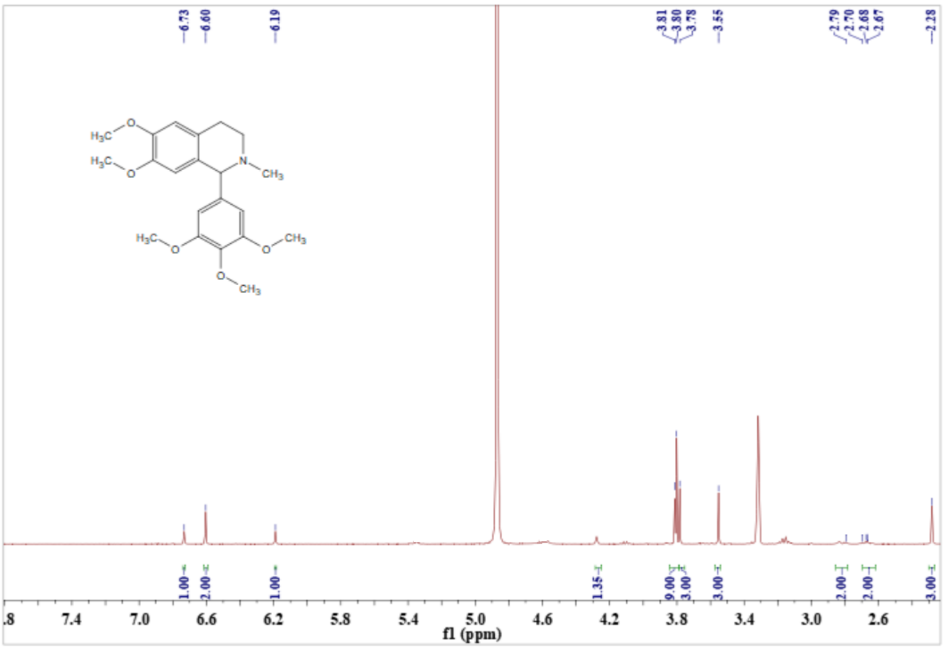

(N)^{1}H NMR spectra of the compound **4** recorded in DMSO.

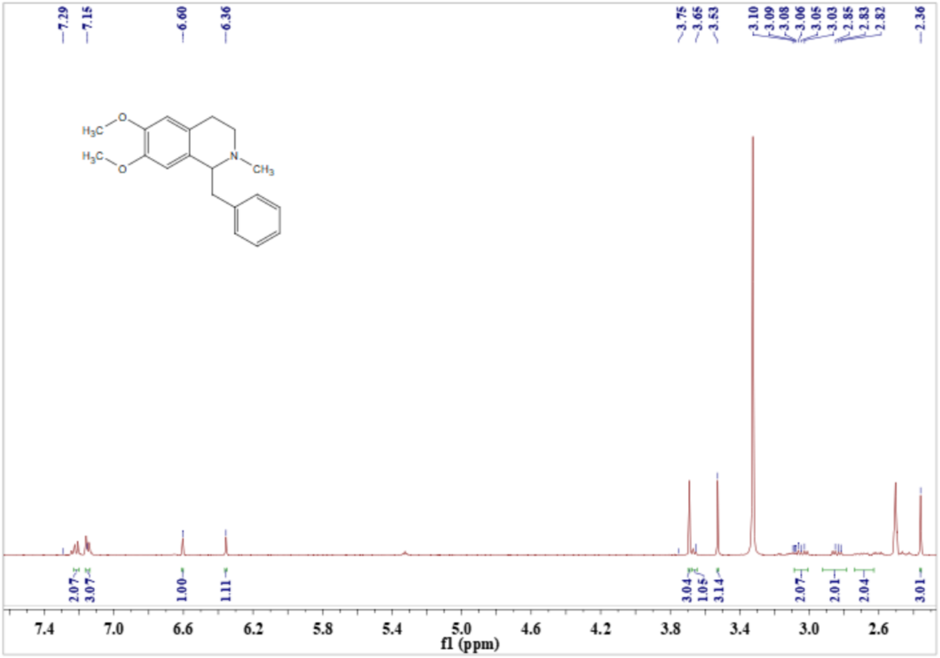

(O)^{1}H NMR spectra of the compound **5** recorded in DMSO.

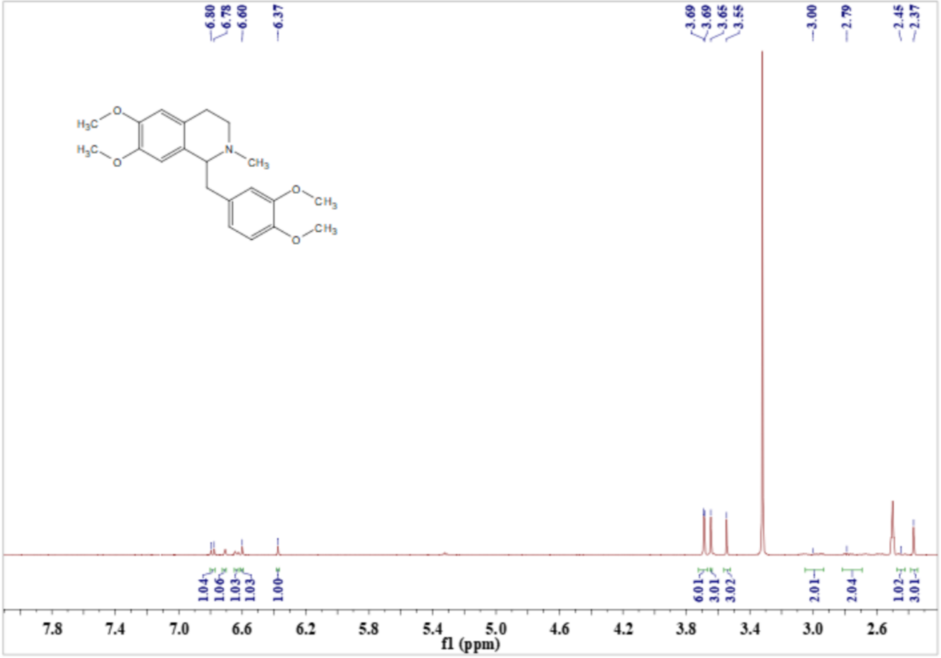

4. Supplementary References

1 J. Sambrook, D. W. Russell and Molecular Cloning: *A Laboratory Manual, 3rd ed.*, Cold Spring Harbor Laboratory Press, New York. 2001.

2 J. Zhu, H. Tan, L. Yang, Z. Dai, L. Zhu, H. Ma, Z. Deng, Z. Tian and X. Qu, *ACS Catal.*, 2017, **7**, 7003-7007.

3 M. Chang, W. Li and X. Zhang, *Angew. Chem. Int. Ed.*, 2011, **50**, 10679-10681.

4 P. Ábrányi-Balogh, T. Földesi and M. Milen, *Monatsh. Chem.*, 2015, **146**, 1907-1912.

5 E.D. José, M. Redondo and M .Wills, *Tetrahedron Asymmetry*, 2010, **21**, 2258-2264.

6 R. Zhu, Z. Xu, W. Ding, S. Liu, X. Shi and X. Lu, *Chin. J. Chem.*, 2014, **32**, 1039-1048.

7 M. Perez, Z. Wu, M. Scalone and T. Ayad, *Eur. J. Org. Chem.*, 2015, **29**, 6503-6514.

8 A. Brossi and S. Teitel, *Tetrahedron.*, 1973, **29**, 31-39.

9 H. Peng, E. Wei, J. Wang, Y. Zhang, L Cheng, H. Ma, Z. Deng and X. Qu, *ACS Chem. Biol.*, 2016, **11**, 3278−3283.

10 L. Chang, J. M. Hagel and P. J. Facchini, *Plant Physiol.*, 2015, **169**, 1127-1140.
